# A Unique B-Family DNA Polymerase Facilitating Error-Prone DNA Damage Tolerance in Crenarchaeota

**DOI:** 10.3389/fmicb.2020.01585

**Published:** 2020-07-23

**Authors:** Xu Feng, Xiaotong Liu, Ruyi Xu, Ruiliang Zhao, Wenqian Feng, Jianglan Liao, Wenyuan Han, Qunxin She

**Affiliations:** ^1^CRISPR and Archaea Biology Research Center, Microbial Technology Institute and State Key Laboratory of Microbial Technology, Shandong University, Qingdao, China; ^2^State Key Laboratory of Agricultural Microbiology, College of Life Science and Technology, Huazhong Agricultural University, Wuhan, China

**Keywords:** *Sulfolobus islandicus*, DNA polymerase, genetic analysis, DNA damage tolerance, Crenarchaeota, Dpo2, Dpo4

## Abstract

*Sulfolobus islandicus* codes for four DNA polymerases: three are of the B-family (Dpo1, Dpo2, and Dpo3), and one is of the Y-family (Dpo4). Western analysis revealed that among the four polymerases, only Dpo2 exhibited DNA damage-inducible expression. To investigate how these DNA polymerases could contribute to DNA damage tolerance in *S. islandicus*, we conducted genetic analysis of their encoding genes in this archaeon. Plasmid-borne gene expression revealed that Dpo2 increases cell survival upon DNA damage at the expense of mutagenesis. Gene deletion studies showed although *dpo1* is essential, the remaining three genes are dispensable. Furthermore, although Dpo4 functions in housekeeping translesion DNA synthesis (TLS), Dpo2, a B-family DNA polymerase once predicted to be inactive, functions as a damage-inducible TLS enzyme solely responsible for targeted mutagenesis, facilitating GC to AT/TA conversions in the process. Together, our data indicate that Dpo2 is the main DNA polymerase responsible for DNA damage tolerance and is the primary source of targeted mutagenesis. Given that crenarchaea encoding a Dpo2 also have a low-GC composition genome, the Dpo2-dependent DNA repair pathway may be conserved in this archaeal lineage.

## Introduction

Multiple DNA polymerases are required in all organisms to duplicate their genomes and repair DNA lesions generated from endogenous compounds and environmental factors. Whereas *Escherichia coli*, the best-studied bacterial model, contains five DNA polymerases, the human genome codes for >15 such enzymes (Vaisman and Woodgate, 2017). These DNA polymerases are of two main types based on their cellular functions: those that are devoted to copy genomes with high fidelity and processivity (replicative polymerases) and those that mainly function in DNA repair (specialized polymerases) (McCulloch and Kunkel, 2008). To date, four families of replicative enzymes are known, that is, Families A, B, C, and D. These replicative polymerases are endowed with the 3′–5′ exonuclease activity that proofreads the synthesized product, and their active centers are only compatible with the Watson–Crick base paring, a characteristic that is essential for copying DNA with high accuracy. Most specialized polymerases capable of translesion DNA synthesis (TLS) belong to the Y-family, and they bypass DNA lesions by accommodating bulky or distorted template at their enlarged active centers. However, there are two exceptions: the bacterial Pol II and the eukaryotic Pol ζ. Although belonging to the B-family DNA polymerases, they are also capable of conducting TLS (Sale, 2013; Chatterjee and Walker, 2017; Yang and Gao, 2018). To date, B-family TLS DNA polymerase has not been identified in Archaea, the third domain of life.

Archaea also code for multiple DNA polymerases as for bacteria and eukaryotes. For example, Sulfolobales, an order of thermophilic acidophiles in Crenarchaeota, have four DNA polymerase genes (*dpo1* through *dpo4*), coding for three B-family DNA polymerases (Dpo1, Dpo2, and Dpo3) and one Y-family polymerase (Dpo4). All four DNA polymerases of *Saccharolobus solfataricus* (Sakai and Kurosawa, 2018) (formerly *Sulfolobus solfataricus* P2) (Zillig et al., 1980) have been expressed as recombinant proteins and analyzed *in vitro*. This reveals that only Dpo1 synthesizes DNA with high processivity and fidelity, and its DNA synthesis is easily blocked by lesions in DNA templates (Gruz et al., 2003; Zhang et al., 2010; Choi et al., 2011; Yan et al., 2017). By contrast, the *S. solfataricus* Dpo4 can bypass various DNA lesions *in vitro* (Boudsocq et al., 2001; Ling et al., 2004; Johnson et al., 2005; Zhang et al., 2006; Fiala et al., 2007; Sherrer et al., 2009; Choi et al., 2011). For Dpo2, the protein carries substitution for key amino acid residues in the PolC motif at its putative active center and exhibits a low primer extension activity in biochemical assays (Rogozin et al., 2008; Choi et al., 2011). As a result, Dpo2 was predicted as an inactive polymerase (Makarova et al., 2014). However, transcriptome analyses reveal that *dpo2* is the only DNA polymerase gene that exhibits DNA damage-inducible expression in three different Sulfolobales organisms including *Sulfolobus acidocaldarius*, *S. solfataricus*, and *Sulfolobus islandicus* (Frols et al., 2007; Gotz et al., 2007; Feng et al., 2018; Sun et al., 2018). This raises an important question as to which DNA polymerase could have an important role in DNA repair in Sulfolobales.

In this work, we aimed to investigate the function of these archaeal DNA polymerases in DNA damage repair with *S. islandicus*, a genetic model for which very versatile genetic tools have been developed (Peng et al., 2017). *S. islandicus* codes for four DNA polymerases (Guo et al., 2011), as for other species in Sulfolobales. Genetic studies on the function of all four DNA polymerase genes in DNA repair unravel that the B-family Dpo2, but not the Y-family Dpo4, is the main polymerase that mediates the tolerance of bulky DNA lesions and is absolutely required for targeted mutagenesis in *S. islandicus*.

## Materials and Methods

### Strains, Cell Growth, and DNA Damage Treatment

The genetic host of *S. islandicus* E233S (Deng et al., 2009) was derived from the original isolate *S. islandicus* REY15A (Contursi et al., 2006). The E233S strain and its deletion derivatives of each DNA polymerase gene (Supplementary Table S1) were grown at 78°C in SCV media (basal media supplemented with 0.2% sucrose, 0.2% Casamino acids, and 1% vitamin solution) (Deng et al., 2009), and uracil was supplemented to 20 μg/ml. pSeSD_*dpo2*/E233S and pSeSD/E233S strains were cultured in ACV medium in which sucrose was replaced with D-arabinose at the same concentration (Peng et al., 2012).

For DNA damage treatment with different mutagenic chemicals, *S. islandicus* strains were grown to an exponential growth phase [absorbance at 600 nm (A600) = 0.2] in SCV/ACV media, and the cultures were treated with a DNA damage agent (1, 2, and 3 μM of NQO or 10 μg/ml of cisplatin) as specified in each experiment. Treated cultures were incubated for 24 h, during which cell samples were taken for A600 measurement, colony-forming unit (CFU) assay, apparent mutation rate assay, flow cytometry analysis, and preparation of cell extracts as previously described (Sun et al., 2018). DNA damage experiments with ultraviolet (UV) light were conducted under the dark condition: 25 ml of culture was transferred into a petri dish of 9 cm in diameter, placed in the center of a CL-1000 Ultraviolet Crosslinker (UVP, Analytik Jena), and irradiated with a setting of 50 J/m^2^ at 254 nm. Treated and untreated cultures were then incubated for 6 h, during which cell samples were taken for CFU assay, the apparent mutation rate assay, and preparation of cell extracts.

### Construction of Deletion Mutants of DNA Polymerase Genes in *S. islandicus*

The CRISPR-based genetic manipulation method developed in our laboratory (Li et al., 2016) was employed to construct deletion mutants of *S. islandicus* for all four DNA polymerase genes (*dpo*), that is, *dpo1*, *dpo2*, *dpo3*, and *dpo4*. Genome-editing plasmids carrying a mini-CRISPR array with a spacer targeting each DNA polymerase gene and a donor DNA flanking the corresponding mutated allele were constructed as follows: (a) spacer DNA fragments were prepared by annealing of two complementary oligos (e.g., KO*dpo2*spf and KO*dpo2*spr). (b) Spacer fragments were individually inserted into the pSe-Rp vector [a vector carrying repeat sequences of CRISPR system (Peng et al., 2015) at the *Sap*I site, giving pAC-*dpo1*, pAC-*dpo2*, pAC-*dpo3*, and pAC-*dpo4* vectors]. (c) Donor DNAs were obtained by the splicing by overlap extension (SOE) PCR (Feng et al., 2018) in which the first set of primers (e.g., KO*dpo2*Larm-F and KO*dpo2*SOE-R) were used to yield the upstream part of the donor DNA, whereas the second set of primers (e.g., KO*dpo2*SOE-F and KO*dpo2*Rarm-R) were used to amplify the downstream part. These two DNA fragments were then spliced together by PCR using the primer combination of the forward primer of upstream fragment and the reverse primer of the downstream fragment, for example, KO*dpo2*Larm-F and KO*dpo2*Rarm-R primers, and this yielded donor DNAs carrying a deletion allele of each DNA polymerase gene. (d) Each donor DNA was inserted into the corresponding pAC-*dpo* vectors at the *Sph*I and *Xho*lI sites, yielding genome-editing plasmid, pGE-*dpo1*, pGE-*dpo2*, pGE-*dpo3*, and pGE-*dpo4*, individually. The plasmids and DNA oligonucleotides employed in this work are listed in Supplementary Table S1 and S2.

Next, each genome-editing plasmid was introduced into *S. islandicus* E233S by electroporation, giving transformants on selective plates. Deletion mutant strains were identified by PCR amplification of each mutant allele using, for example, KO*dpo2* F/KO*dpo2* R and *dpo2*inner F/*dpo2*inner R primers and verified by DNA sequencing of deletant alleles. Finally, genome editing plasmids were cured from the mutants by counterselection for *pyrEF* marker using uracil and 5-FOA, yielding Δ*dpo2*, Δ*dpo3*, and Δ*dpo4* for further experiments.

### Immunoblotting Analysis

Ten milliliters of NQO/UV-treated or reference cultures were collected by centrifugation and resuspended in 150 μl of TBST buffer (50 mM of Tris–HCl, 100 mM of NaCl, and 0.1% Tween-20, pH 7.6). Cells in the cell suspensions were then disrupted by sonication using a JY92-IIN ultrasonic homogenizer (Scientz Biotechnology), giving cell extracts for each cell sample. Proteins in each sample (10 μg) were separated according to their sizes on a 10% gel by sodium dodecyl sulfate–polyacrylamide gel electrophoresis (SDS-PAGE). Proteins in the gel were then transferred onto a nitrocellulose membrane using a Trans-Blot Semi-Dry Transfer Cell (Bio-Rad). For immunoblotting, the membrane was first immersed in 5% skim milk blocking agent for 60 min and then incubated with individual primary antibody for another 60 min and finally with corresponding secondary antibody for 60 min. Hybridization signals were generated on the membrane using the ECL western blot substrate (Millipore) and visualized by exposure of the membrane to an X-ray film.

### Determination of Cell Viability

Cells in 1 ml of NQO/UV-treated or untreated reference cultures were harvested by centrifugation. Cell pellets were resuspended in 1 ml fresh SCV medium from which 100 μl of cell suspension was taken out and used for a serial dilution to 10^–5^; 100 μl of diluted cell sample was plated onto an SCV plate supplemented with 20 μg/ml uracil. After incubation for 7 days, colonies that appeared on plates were counted, and the resulting data were used for the calculation of survival rates, which were expressed as the percentage of viable cells in NQO/UV-treated cultures relative to those in the corresponding untreated references.

### Determination of Apparent Mutation Rates and Mutation Spectrum

Cells in drug/UV-treated or reference cultures were collected by centrifugation. After being resuspended in fresh SCV medium, 1,000 μl of cell suspension was plated on SCV plates containing 20 μg/ml of uracil, 160 μM of 6-methylpurine (6-MP) and 0.5 mM of GMP (6-MP plates), using a two-layer plating technique as previously described (Deng et al., 2009). In the meantime, an aliquot of the cell suspension was serially diluted, and the diluted cell samples were plated on drug-free SCV plates. After incubation for 7 days, colonies that appeared on plates were counted and the resulting data were used for the calculation of the apparent mutation rate.

To identify mutations of the *apt* gene in 6-MP^r^ mutants, ∼120 colonies for each sample were randomly picked up on 6-MP plates from two independent experiments and used as templates for PCR amplification of the marker gene using the primer set of Sisapt F/Sisapt R. The resulting PCR products were sent for DNA sequencing in BGI. Sequencing results were then aligned with the genomic region containing the putative promoter and coding sequence of the *apt* gene using BioEdit 7.0.9.0 (Hall, 1999). Mutated sites of the *apt* gene were identified and classified into different types based on the nature of the mutations, and mutations identified for each mutant strain were compiled together, yielding the mutation spectrum in which hotspots could be readily identified.

### Flow Cytometry Analysis

Flow cytometry of *S. islandicus* cells was conducted as previously described (Han et al., 2017). Briefly, NQO-treated or reference samples were fixed by 70% ice-cold ethanol individually for an hour on ice. Cells were harvested by centrifugation at 8,000 rpm for 4 min. After being washed twice with 10 mM of Tris–HCl, 10 mM of MgCl_2_, pH 8.0 buffer, cell pellets were resuspended in the staining buffer containing 40 μg/ml of ethidium bromide (Sigma) and 100 μg/ml of mithramycin A (Apollo Chemical, Tamworth, United Kingdom) and incubated on ice for 30 min. Stained cell samples were then analyzed by flow cytometry with an Apogee A40 (Apogee System, Hertfordshire, United Kingdom) equipped with a 405-nm laser. A dataset of at least 30,000 cells was collected for each sample.

### Phylogenetic Analysis

Dpo2 homologs were identified using National Center for Biotechnology Information (NCBI) Blast, with the protein sequence of the *S. islandicus* REY 15A Dpo2 as a query. 16S rDNA sequences of crenarchaeotal species were retrieved from NCBI. Phylogenetic analysis was performed on the Phylogeny.fr platform (Dereeper et al., 2008). Specifically, protein sequences of Dpo2 homologs and 16S rDNA sequences were first aligned individually using the MUSCLE program (v3.5). The poorly aligned regions were then removed by Gblocks program (v0.91b) using the default setting. The phylogenetic trees were constructed using PhyML program (v3.0) with default setting. At last, the trees were visualized using the TreeDyn program or the Interactive Tree Of Life (iTOL) webserver (Letunic and Bork, 2019).

## Results

### Expression of Dpo2 Protein Is Tightly Controlled During DNA Damage Treatment

**FIGURE 1:**
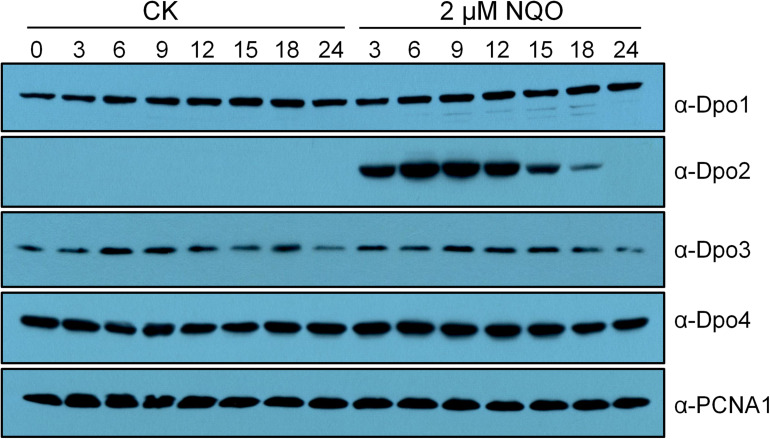
Expression profiles of four DNA polymerases in *Sulfolobus islandicus*. Exponential growth cultures (A600 = 0.2) of the wild-type (WT) strain, *S. islandicus* E233S was treated with DNA damage agent (2 μM of NQO) or untreated (CK), and these cultures were then grown for 24 h during which cell samples were taken at indicated time points (hours) and used for preparation of cell extracts. Proteins in the cell extracts (10 μg) were fractionated by sodium dodecyl sulfate–polyacrylamide gel electrophoresis (SDS-PAGE), and DNA polymerases were identified by western blotting and hybridization using antibodies raised against each DNA polymerase. PCNA1, a subunit of the archaeal replication sliding clamp, serving as a loading control.

Previous transcriptome studies showed that among the four DNA polymerase genes, only *dpo2* undergoes DNA damage response (DDR) expression in *S. solfataricus*, *S. acidocaldarius*, and *S. islandicus* (Frols et al., 2007; Gotz et al., 2007; Feng et al., 2018; Sun et al., 2018). To investigate the expression of all four DNA polymerases at the protein level, the wild-type (WT) strain of *S. islandicus* was incubated with NQO, a mutagen that predominately induces helix-distorting bulky adducts (Bailleul et al., 1989), in a time course of 24 h. Cell samples were taken at different time points during the incubation and used for western blotting analysis. We found that (a) no apparent fluctuation of protein content was observed for Dpo1, Dpo3, and Dpo4 during the drug treatment and that (b) the Dpo2 protein, although present at a level below the detection limit before the drug addition, attained the highest level at 6 h post NQO addition (p.n.a.), with the plateau level maintained for 9 h in the cell before dropping down, and became barely detectable at 24 h p.n.a. (Figure 1). These results suggested that the content of Dpo2 could be correlated to the level of DNA damage in *S. islandicus.*

To gain a further insight into the Dpo2 regulation, relative Dpo2 contents were compared in *S. islandicus* cells treated with three different concentrations of NQO (1, 2, or 3 μM), again in a time course window of 24 h. We found that the drug triggered differential expression of Dpo2: upon the treatment with 1 μM of NQO, the plateau level of the enzyme was delayed for 3 h in the cells; when the maximum level was attained, it was ∼4-fold lower than that in the cells treated with the two higher doses, that is, 2 and 3 μM of NQO (Supplementary Figure S1). In addition, the NQO-mediated growth inhibition was also dose dependent: although the addition of 1 μM of NQO yielded little influence on the change of the optical density of the cultures during incubation, the drug moderately impaired the culture growth at 2 μM, whereas much stronger growth inhibition was observed for the cultures treated with 3 μM of NQO (Supplementary Figure S1). Strikingly, the plateau periods of Dpo2 expression were also correlated to the drug concentrations in NQO-treated cultures, being ∼6, 9, and 12 h for cultures treated with 1, 2, and 3 μM of NQO, respectively (Supplementary Figure S1). Because the amount of DNA lesion increases along with the elevation of drug concentration, these results indicated that Dpo2 expression is regulated by DNA damage: the gene expression is to be activated in the cell upon the occurrence of DNA lesions and to be deactivated upon the complete removal of the lesions from its genomic DNA. To this end, these data suggested that Dpo2 could be important for DNA damage repair in *S. islandicus*.

### Dpo2 Overexpression Enhances Cell Viability Upon DNA Damage Treatment

**FIGURE 2:**
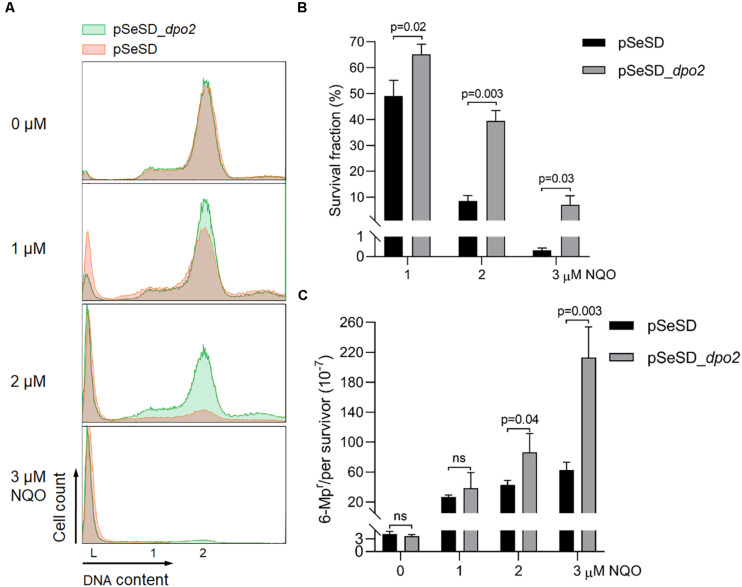
Dpo2 overexpression elevates DNA damage tolerance of *Sulfolobus islandicus* at expense of mutagenesis. **(A)** DNA content profile. The *X*-axis refers to DNA content, and the *Y*-axis indicates the cell numbers counted. Cell populations with one chromosome (1), two chromosomes (2), and DNA-less cells (L) are marked. **(B)** Survival rates. Colony-forming units of untreated sample (0 μM) were arbitrary assigned to 100% for each strain (pSeSD, 0 μM; and pSeSD_*dpo2*, 0 μM), with which the survival fraction of NQO-treated sample was calculated. **(C)** Apparent mutation rate. Values were obtained by dividing the number of 6-methylpurine resistant (6-MP^r^) colonies with the number of colonies formed on the corresponding 6-MP-free plates. The *dpo2*-overexpression strain (pSeSD_*dpo2*) and its reference (pSeSD) were grown in the absence or presence of NQO (0, 1, 2, and 3 μM) for 24 h. Cells were then collected and employed for flow cytometry and determination of colony formation and apparent mutation rates. At least three independent experiments were performed with the standard deviation shown in the error bar. Multiple *t*-tests or one-way ANOVA was performed in the statistical analysis, with *p*-values indicated in the bar graph.

To investigate the function of Dpo2, the encoding gene in *S. islandicus* was cloned to pSeSD (Peng et al., 2012), giving the expression plasmid pSeSD-*dpo2* in which the *dpo2* expression is subjected to arabinose induction. The expression plasmid and the cloning vector were introduced into the WT strain (E233S) by transformation, giving pSeSD-*dpo2*/E233S and pSeSD/E233S, respectively. These strains were then grown in ACV, a medium with D-arabinose as the sole carbon source. When their cell density reached A600 = 0.2, NQO was added to the cultures at 0, 1, 2, and 3 μM individually. The cultures were further incubated for 6 h, a time point at which Dpo2 was expected to attain a plateau expression level in all NQO-treated cultures (Supplementary Figure S1). Cells were then collected and used for preparation of cell extracts. Dpo2 in the cell extracts was subsequently detected by western blotting and hybridization using antisera raised against Dpo2 or a penta-histidine tag. We found that Dpo2 was expressed to a very high level in pSeSD-*dpo2*/E233S cells grown in the ACV medium, which is consistent with the feature of arabinose-inducible expression from the expression vector (Peng et al., 2012). In contrast, the enzyme was expressed to an undetectable level in the reference cells (pSeSD/E233S) (Supplementary Figure S2). Because the two cultures showed very similar cell cycle profiles (Figure 2A), these results indicated that the occurrence of a large amount of Dpo2 in the cell does not influence the DNA replication and cell cycle progression of *S. islandicus*.

However, during DNA damage treatment, the cells containing large amounts of pre-expressed Dpo2 proteins showed a less extent of chromosome degradation and consequently a lower rate of cell death as revealed by flow cytometry (Figure 2A). To verify the data, cultures of Dpo2-overexpressing strain and its reference were plated for CFU to determine their survival rates. As shown in Figure 2B, compared with the reference, which has 49.1, 8.6, and 0.3% survival fraction post 1, 2. and 3 μM of NQO treatment individually, *dpo2*-overexpression cells showed a higher survival rate in the presence of all three levels of NQO, being 64.2, 39.4, and 7.1%, respectively. The elevation of cell survival rate by Dpo2 overexpression was positively related to the drug doses, being 1. 3-, 4. 6-, and 22.9-fold for treatments with 1, 2, and 3 μM of NQO, respectively. These data indicated that Dpo2 overexpression greatly enhanced the resistance of *S. islandicus* to NQO.

To investigate if Dpo2 overexpression could influence targeted mutagenesis in *S. islandicus*, apparent mutation rates were determined for the cells in the NQO-treated cultures as well as the corresponding untreated references, using the *apt*-based forward (loss-of-function) mutation assay as previously described (Zhang et al., 2016). As shown in Figure 2C, spontaneous mutation rates of the two strains were very similar in the absence of NQO, indicating that Dpo2 polymerase did not exert any influence on the replication fidelity of the organism even in the presence of a large excessive amount (∼20-fold of the NQO-induced level; see Supplementary Figure S2). However, in the presence of NQO, the mutation rate of the marker gene was differentially elevated in these two strains: 1. 6-, 2. 3-, and 3.9-fold higher mutation rates were observed for the cultures of the overexpression strain treated with 1, 2, and 3 μM of NQO, respectively, compared with their corresponding references. These results indicated that Dpo2 facilitates targeted mutations in *S. islandicus* and may mediate the tolerance of DNA lesions induced by external mutagen*s*.

### Both Dpo2 and Dpo4 Contribute to NQO Lesion Tolerance in *S. islandicus*

To investigate the function of each DNA polymerases in the DNA damage tolerance in *S. islandicus*, we attempted to construct gene deletion mutants for each DNA polymerase gene as described in the section “Materials and Methods”. Three were obtained, including Δ*dpo2*, Δ*dpo3*, and Δ*dpo4*, but construction of a *dpo1* gene deletant constantly failed in this archaeon. All three mutants were then checked for the presence of the four DNA polymerases by western analysis, with antisera raised against each DNA polymerase. As shown in Supplementary Figure S3, the absence of individual DNA polymerase in each mutant was confirmed by the lack of the corresponding hybridization signal. These results indicated that whereas *dpo1* encodes the main replicase that is essential for cell viability, *dpo2*, *dpo3*, and *dpo4* code for non-essential DNA polymerases.

**FIGURE 3:**
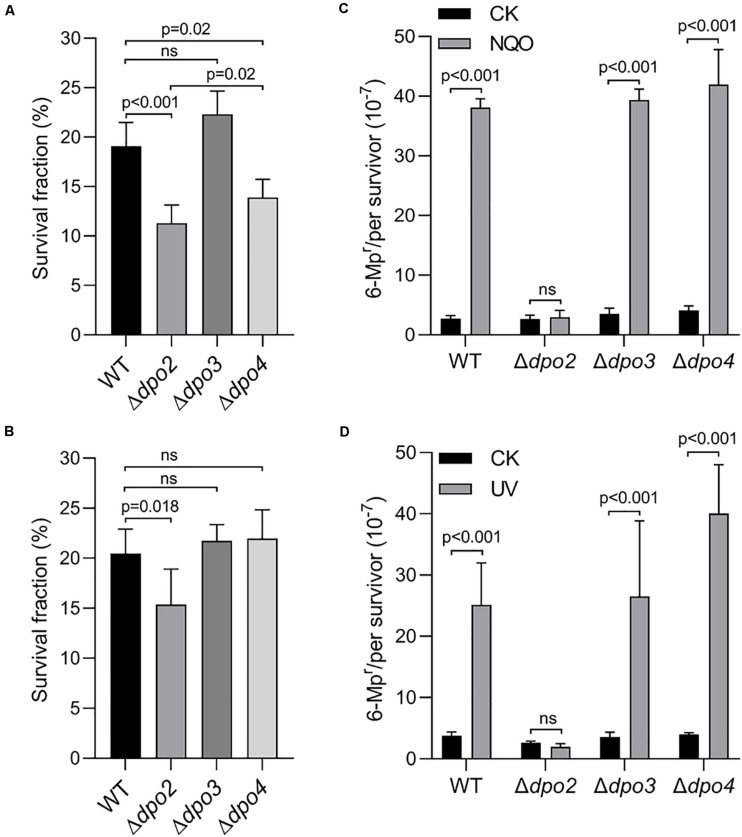
Contributions of accessory DNA polymerases to cell survival and targeted mutagenesis upon NQO and ultraviolet (UV) treatment. **(A,C)**. NQO treatment. **(B,D)**. UV-C irradiation. For NQO treatment, exponential cultures were grown in the presence of 2 μM of NQO for 24 h; and for UV irradiation, the cultures were exposed to 50 J/m^2^ of UV-C light and incubated for 6 h for recovery. Then, untreated and treated cultures were plated for colony-forming units (CFU). CFU of untreated sample (CK) was defined as 100% for each strain, with which the survived fraction of cells in NQO/UV-treated samples was calculated. These samples were also plated on nutrient medium plates containing 6-methylpurine for determination of mutation rates, as described in Figure 2. At least three independent experiments were performed with the standard deviation shown in the error bar. Multiple *t*-tests or one-way ANOVA was performed in the statistical analysis, with *p*-values indicated in the bar graph.

Next, the three mutants were studied for the contribution of each non-essential DNA polymerase to cell survival and mutagenesis. Again, NQO was used as the DNA damage agent for testing DNA damage tolerance. We found that loss of *dpo2* and *dpo4* reduced the cell viability of their NQO-treated cultures by ∼42 and ∼26% individually, relative to that of the reference strain (WT), and loss of *dpo3* did not exhibit any influence because the treated Δ*dpo3* and WT cultures showed very similar cell viability (Figure 3A). These results indicated that whereas both Dpo2 and Dpo4 confer NQO resistance to *S. islandicus* cells, Dpo3 does not play a role in the correction of NQO-induced lesions.

DNA damage tolerance in these strains was further evaluated by the forward mutation assay with the *apt* marker gene. Whereas the mutation rate of Δ*dpo2* remained more or less constant between treated and untreated cultures, treated cultures of the remaining three strains showed >10-fold increases in the mutation rate, relative to their untreated references (Figure 3C). These results indicated that Dpo2 is responsible for the mutagenic tolerance of NQO-induced lesions in *S. islandicus*.

### Dpo4 Contributes to Untargeted Mutations

**FIGURE 4:**
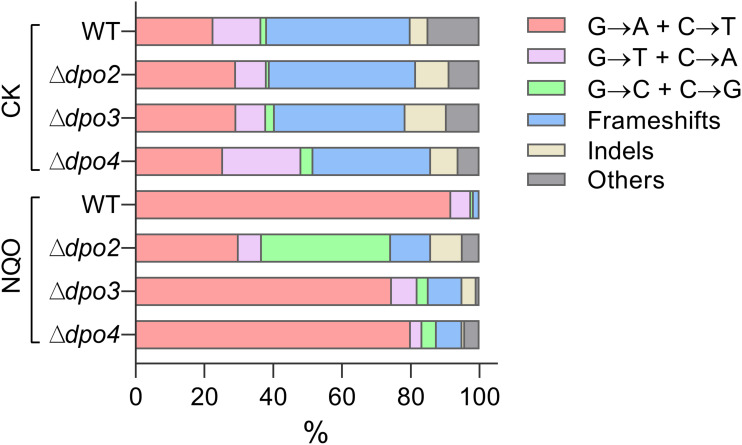
Targeted and untargeted mutation spectra of *Sulfolobus islandicus* wild-type strain and DNA polymerase gene mutants. Untargeted mutation spectra were obtained from the cultures grown in the medium lacking any external mutagen. Targeted mutation spectra were from cultures grown in the medium containing 2 μM of NQO. The length of each bar graph for each category of mutation is proportional to the corresponding% of mutation presented in Table 1. Frameshifts: 1–2 bp of indels with a majority of ± 1 frameshifts. Indels: > 2 bp of indels. Other: other base substitutions.

To gain an understanding of the generation of untargeted mutations in *S. islandicus*, a total of 120 colonies were randomly picked up on forward mutation assay plates of the WT strain and each *dpo* mutant and used as templates for PCR amplification of the *apt* gene. The resulting PCR products were sequenced to reveal spontaneous mutations that occurred in the marker gene in these strains. The identified mutations were categorized into six different types: three main types of base substitutions (BSs) (including GC → AT, GC → TA, and GC → CG substitutions), other BS mutations, frameshifts, and other insertions and deletions (indels). Their occurrence frequencies in the four different strains were summarized in Table 1 and illustrated in Figure 4. We found that profiles of untargeted mutations were very similar for WT, Δ*dpo2*, and Δ*dpo3*, whereas Δ*dpo4* showed a reduction in frameshift mutations (cf. 58% for WT vs. 42% for Δ*dpo4*), indicating that Dpo4 contributes to the formation of untargeted indels in this organism.

**TABLE 1:** Spontaneous and NQO-induced mutations at the *apt* locus of WT and *dpo* mutants.

Type of mutation	WT CK	WT NQO	Δ*dpo2* CK	Δ*dpo2* NQO	Δ*dpo3* CK	Δ*dpo3* NQO	Δ*dpo4* CK	Δ*dpo4* NQO
**Base substitutions**	53.1%^a^ (61^b^)	97.5% (117)	47.8% (54)	79.2% (95)	50% (58)	86.1% (105)	57.9% (66)	91.7% (110)
**Transitions**								
GC–AT	22.6% (26)	91.7% (110)	29.2% (33)	30% (36)	29.3% (34)	74.5% (91)	25.4% (29)	80% (96)
AT–GC	4.4% (5)	0	3.5% (4)	3.3% (4)	2.6% (3)	0	0.9% (1)	1.7% (2)
**Transversions**								
GC–TA	13.9% (16)	5.8% (7)	8.9% (10)	6.7% (8)	8.6% (10)	7.4% (9)	22.8% (26)	3.3% (4)
GC–CG	1.7% (2)	0.8% (1)	0.9% (1)	37.5% (45)	2.6% (3)	3.3% (4)	3.5% (4)	4.2% (5)
AT–CG	1.7% (2)	0	3.5% (4)	0.8% (1)	2.6% (3)	0	0.9% (1)	0
AT–TA	8.7% (10)	0	1.8% (2)	0.8% (1)	4.3% (5)	0.8% (1)	4.4% (5)	2.5% (3)
**Indels**	46.9% (54)	1.7% (2)	52.2% (59)	20.8% (25)	50% (58)	13.9% (17)	42.1% (48)	8.3% (10)
**Frameshifts**	41.7% (48)	1.7% (2)	42.5% (48)	11.7% (14)	37.9% (44)	9.8% (12)	34.2% (39)	7.5% (9)
Minus 1 bp	17.4% (20)	0.8% (1)	14.2% (16)	6.7% (8)	18.1% (21)	6.6% (8)	18.4% (21)	2.5% (3)
Plus 1 bp	24.4% (28)	0.8% (1)	27.4% (31)	4.2% (5)	19.8% (23)	3.3% (4)	14.0% (16)	5% (6)
Plus/minus 2 bp	0	0	0.9% (1)	0.8% (1)	0	0	1.8% (2)	0
**Large Indels**	5.2% (6)	0	9.7% (11)	9.2% (11)	12% (14)	4.1% (5)	7.9% (9)	0.8% (1)
Insertions > 2 bp	2.6% (3)	0	4.4% (5)	5.8% (7)	9.5% (11)	4.1% (5)	5.3% (6)	0.8% (1)
Deletions > 2 bp	2.6% (3)	0	5.3% (6)	3.3% (4)	2.6% (3)	0	2.6% (3)	0
**Total**	115	120	113	120	116	122	114	120

*WT, wild type. ^*a*^% of total. ^*b*^Number of occurrences.*

### Dpo2 Is Responsible for Mediating UV-Induced Lesion Tolerance in *S. islandicus*

To investigate which *dpo* gene(s) could mediate DNA damage tolerance to UV irradiation in this archaeon, one of the most common mutagens employed for DNA repair study in different model organisms (Friedberg et al., 2005), these *dpo* deletants were treated with 50 J/m^2^ of UV-C light (254 nm). Irradiated and untreated cultures were then incubated for 6 h and analyzed for cell viability and mutation rates, as described above for NQO experiments. As shown in Supplementary Figure S4, UV-C light also strongly induced Dpo2 expression, but it had little influence on the expression of the remaining three DNA polymerases. As in the NQO experiments, while UV-C light did not facilitate the mutation rate in Δ*dpo2*, the DNA damage agent stimulated mutation rates in all remaining strains, being 6. 6-, 7. 5-, and 9.9-fold for WT, Δ*dpo3*, and Δ*dpo4*, respectively (Figure 3D). In addition, only the deletion of *dpo2* causes a reduction in the survival rate (Figure 3B). These results indicated that only Dpo2 can facilitate UV-damage tolerance, and the tolerance occurred at the expense of an elevated level of mutagenesis in this archaeon.

### Dpo2 Shapes Mutation Spectra Induced by Helix-Distorting Lesions

Next, NQO-induced mutation spectra were analyzed in detail for treated cultures of all tested strains. As summarized in Table 1 and illustrated in Figure 4, NQO induced > 4-fold increase in the fraction of GC → AT transitions in WT strain (cf. 23% in untreated cells vs. 92% in treated cells), whereas the elevation was completely abolished in Δ*dpo2* because the GC → AT alteration rate did not change (cf. 29% vs. 30%), and furthermore, major changes were observed for Δ*dpo3* and Δ*dpo4* (cf. 29% vs. 75% for Δ*dpo3*; 25% vs. 80% for Δ*dpo4*). Taken together, we concluded that Dpo2 may have mediated GC to AT transitions on NQO-induced lesions during error-prone DNA synthesis.

**FIGURE 5:**
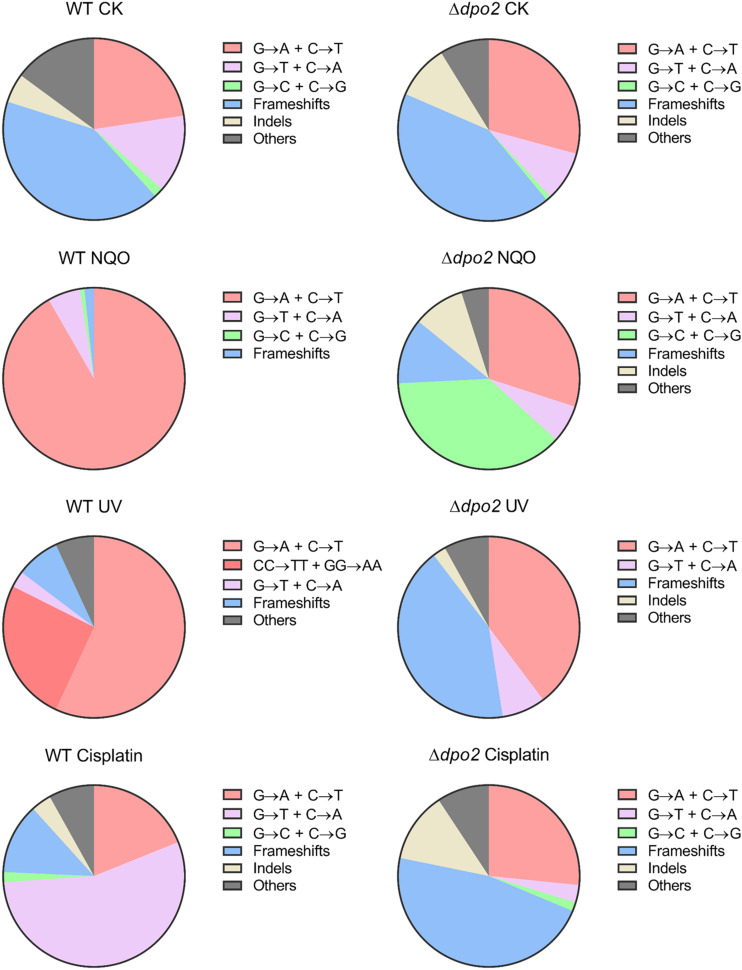
Pie charts of different types of mutations induced by different mutagens in *Sulfolobus islandicus* wild-type (WT) and Δ*dpo2*. Untargeted mutation spectra were obtained from the cultures grown in the absence of any mutagen. Targeted mutation spectra were from cultures treated with 2 μM of NQO, 50 J/m^2^ of UV-C and 10 μg/ml of cisplatin, individually. The fractions shown in each pie chart are converted from mutation percentages of each category presented in Table 2. WT, *S. islandicus* E233S; Δ*dpo2*, *S. islandicus dpo2* deletion mutant. Frameshifts: 1–2 bp of indels with a majority of ±1 frameshifts. Indels: >2 bp of indels. Others: other base substitutions.

To gain a further insight into the Dpo2-mediated DNA damage tolerance, we analyzed spontaneous mutation spectra of Δ*dpo2* and WT as well as their targeted ones induced by UV-C light and cisplatin, which produce helix-distortion lesions including linked pyrimidine dimers and crosslinked strand lesions, respectively (Friedberg et al., 2005). Untargeted and targeted mutation data were summarized in Table 2, with the corresponding mutation spectrum profiles illustrated in Figure 5. We found that these mutagens induced different types of dominant mutations in the WT *S. islandicus* cells: (a) for UV irradiation, the GC → AT transition was elevated for 34% (cf. 23% for untreated cells vs. 57% for treated cells), whereas the tandem BSs of CC to TT and GG to AA were augmented for 25% (0 vs. 25%); (b) the cisplatin-mediated GC → TA transversion was elevated for 40% (14% vs. 55%). Given that these targeted mutations were absent from Δ*dpo2* cells treated with the same mutagens (Table 2 and Figure 5), we concluded that Dpo2 is solely responsible for the generation of targeted mutations in *S. islandicus*. Together, our data indicated that *dpo2* codes for an error-prone DNA polymerase that bypasses various helix-distorting lesions in *S. islandicus*, mediating GC → AT/TA mutagenesis in these processes.

**TABLE 2:** Spontaneous and DNA damage-induced mutations at the *apt* locus of WT and *dpo2* mutant.

Type of mutation	WT CK	WT NQO	WT UV	WT cisplatin	Δ*dpo2* CK	Δ*dpo2* NQO	Δ*dpo2* UV	Δ*dpo2* cisplatin
**Base substitutions**	53.1%^a^ (61^b^)	97.5% (117)	92.2% (94)	83.8% (93)	47.8% (54)	79.2% (95)	54.2% (45)	40.6% (26)
**Transitions**								
GC–AT	22.6% (26)	91.7% (110)	56.9% (58)	18.9% (21)	29.2% (33)	30% (36)	41% (34)	26.6% (17)
GG–AA/CC–TT	0	0	25.5% (26)	0	0	0	0	0
AT–GC	4.4% (5)	0	3.9% (4)	1.8% (2)	3.5% (4)	3.3% (4)	2.4% (2)	4.7% (3)
**Transversions**								
GC–TA	13.9% (16)	5.8% (7)	2.9% (3)	55% (61)	8.9% (10)	6.7% (8)	4.8% (4)	3.1% (2)
GC–CG	1.7% (2)	0.8% (1)	0	1.8% (2)	0.9% (1)	37.5% (45)	0	1.6% (1)
AT–CG	1.7% (2)	0	0	0	3.5% (4)	0.8% (1)	2.4% (2)	3.1% (2)
AT–TA	8.7% (10)	0	2.9% (3)	6.3% (7)	1.8% (2)	0.8% (1)	3.8% (3)	1.6% (1)
**Indels**	47% (54)	1.7% (2)	7.8% (8)	16.2% (18)	52.2% (59)	20.8% (25)	45.8% (38)	59.4% (38)
**Frameshifts**	41.7% (48)	1.7% (2)	7.8% (8)	12.6% (14)	42.5% (48)	11.7% (14)	43.4% (36)	46.9% (30)
Minus 1 bp	17.4% (20)	0.8% (1)	1% (1)	9% (10)	14.2% (16)	6.7% (8)	18% (15)	21.9% (14)
Plus 1 bp	24.4% (28)	0.8% (1)	6.9% (7)	2.7% (3)	27.4% (31)	4.2% (5)	24.1% (20)	21.9% (14)
Plus/minus 2 bp	0	0	0	0.9% (1)	0.9% (1)	0.8% (1)	1.2% (1)	3.1% (2)
**Large indels**	5.2% (6)	0	0	3.6% (4)	9.7% (11)	9.2% (11)	2.4% (2)	12.5% (8)
Insertions > 2 bp	2.6% (3)	0	0	0	4.4% (5)	5.8% (7)	2.4% (2)	9.4% (6)
Deletions > 2 bp	2.6% (3)	0	0	3.6% (4)	5.3% (6)	3.3% (4)	0	3.1% (2)
**Total**	115	120	102	111	113	120	83	64

*WT, wild type. ^*a*^% of total. ^*b*^Number of occurrences.*

### *Sulfolobus islandicus* Dpo2 Is Conserved in Crenarchaea With a Low-GC Composition Genome

The concurrence of the AT-rich genome and the targeted GC → AT/TA conversions by Dpo2 in *S. islandicus* is very interesting because it raises a question if the mutagenic DNA damage tolerance by Dpo2 could play a role in sculpting genomic base composition of these archaea during evolution. To test that, we identified Dpo2 homologs (designated as PolB2s) in representative species belonging to Sulfolobales, Desulfurococcales, Acidilobales, and Fervidicoccales (Supplementary Table S3). Multiple sequence alignments of these PolB2s and replicative PolBs revealed that the former are devoid of a recognizable proofreading domain and carry a substitution for the first aspartate residue in the PolC motif (YGDTDS, Supplementary Figures S5, S6). Then, sequences of these crenarchaeotal PolB2s were employed for construction of the protein tree. In the meantime, 16S rDNA sequences of crenarchaeotal species were also used for the phylogenetic analysis. The resulting trees are shown in Supplementary Figures S7, S8. We found that the topology of Dpo2-based tree is highly similar to that of the 16S rDNA-based tree, suggesting that Dpo2 is inherited vertically in Crenarchaeota, although it only commonly occurs in Sulfolobales and shows a scattered distribution in other orders of Crenarchaeota.

**FIGURE 6:**
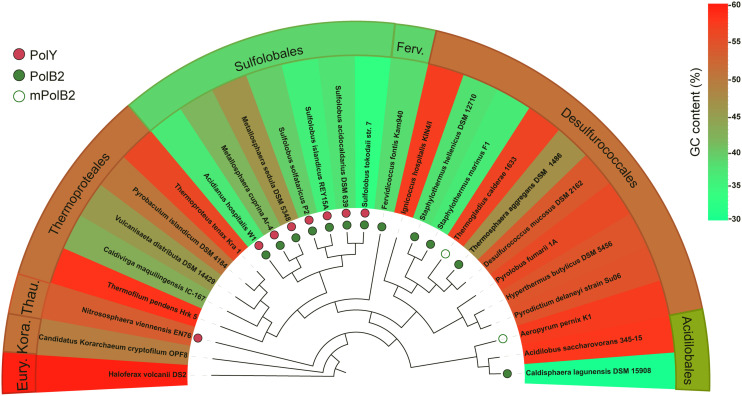
Correlation of PolB2 and GC-poor genomes in Crenarchaeota. DNA sequences of 16S rRNA genes of different crenarchaeotal species were retrieved from the GenBank database and used for construction of phylogenetic trees using the Phylogeny.fr platform. The presence of crenarchaeotal PolB2s and PolYs was annotated on the phylogenetic tree, whereas their genome GC contents are color coded for each species. Empty circles indicate variant PolB2s (mPolB2) carrying substitutions in the active site or sequence insertions at important regions, whereas filled green circles indicate canonical PolB2s. PolYs are shown with filled red circles. A color key in the upper right part indicating genome GC composition (%). Eury., Thau., and Kora. represent Euryarchaeota, Thaumarchaeota, and Korarchaeota phyla, respectively. The background color for the annotations at outer sphere reflects the average GC content of representative species in the corresponding order.

Next, the phyletic distribution of Dpo2 was overlaid onto the 16S rDNA-based tree in which their genome GC contents are color coded for each species (Figure 6), and this revealed that organisms in the Sulfolobales order encode a PolB2 of similar sizes [540–582 amino acids (aa)], sharing 71–96% sequence similarity to the *S. islandicus* Dpo2 (Supplementary Table S3), whereas PolB2 polymerases encoded in organisms of other crenarchaeotal orders, including Desulfurococcales, Acidilobales, and Fervidicoccales, show a low sequence similarity (46–59%), and this is consistent with the phylogenetic relation between these crenarchaeal organisms. Most of the PolB2-encoding organisms have a low GC content, as highlighted in green or yellow green in Figure 6. For example, two closely related organisms in the Acidilobales order, *Acidilobus saccharovorans* 345-15 and *Caldisphaera lagunensis* DSM15908, are very different in genomic GC composition: the former has a GC content of 57.2%, whereas the latter, 30%. Only the GC-poor genome, namely, *C. lagunensis*, codes for a Dpo2 homolog. Nevertheless, there are two exceptions: *Aeropyrum pernix* K1 and *Thermogladius calderae* 1633 encode a variant PolB2 (mPolB2) enzyme, but their genomes have a high GC composition. We noticed that the *Aeropyrum* mPolB2 proteins carry additional mutations in YxGGA motif and PolA motif at their active center (Supplementary Figure S9), which are implicated in DNA binding and sugar binding (Truniger et al., 1996; Kropp et al., 2017), whereas an insertion of 12 amino acids is present in the connecting region between N-terminal and polymerase domain of the *T. calderae* mPolB2 (Supplementary Figure S6). Together, our data not only provides evidence for a Dpo2-driven generation of genomes with low GC composition but also reveals functional diversification of this fascinating B-family DNA polymerase.

## Discussion

**FIGURE 7:**
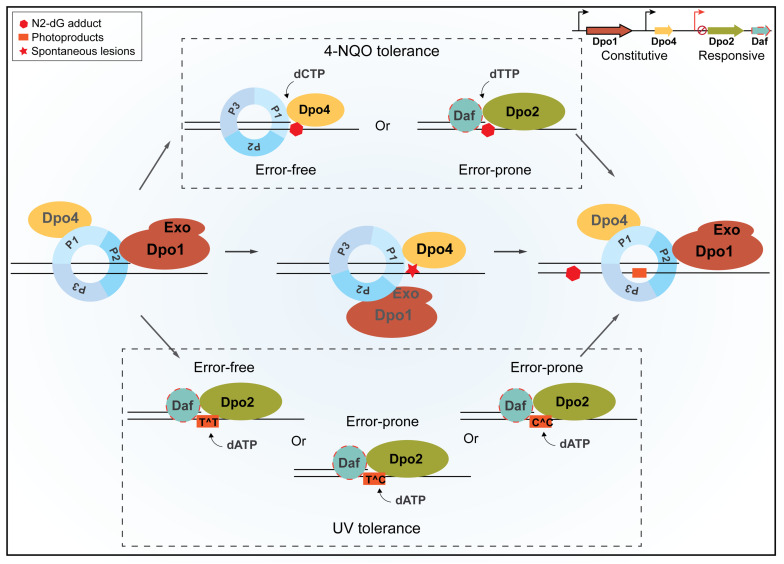
A schematic view of housekeeping and damage-inducible translesion DNA synthesis (TLS) in *Sulfolobus islandicus*. Dpo1 is mainly responsible for the faithful DNA synthesis under normal growth. Upon encountering a lesion in the template, for example, 8-oxodG, polymerase switch occurs, in which the primer termini are transferred to Dpo4 for the error-free bypass. Nevertheless, the dynamic interaction during polymerase switch between Dpo1 and Dpo4 may facilitate nascent strand slippage at sites of mononucleotide runs of adenine, leading to +1 frameshift mutations by Dpo4. In the presence of external mutagens, the expression of Dpo2 is activated probably along with putative Dpo2-associated factor(s) (Daf). Daf may either bring Dpo2 onto the site of lesions and/or activate the activity of the enzyme. NQO-induced lesions can be bypassed either by Dpo4 or by Dpo2, leading to error-free and error-prone bypass, respectively. Tolerances of UV-induced photoproducts at dipyrimidine sites are mainly, if not exclusively, performed by Dpo2, which could generate a single GC to AT transition at a TC/CT site and tandem mutations at CC sites but could lead to the faithful replication of TT photoproducts. Once the lesion bypass was completed, the primer termini are transferred back to Dpo1, and the regular replication resumes. P1, P2, and P3 represent three subunits of the *Sulfolobus* PCNA trimer, and Exo refers to the exonuclease domain of Dpo1.

Sulfolobales organisms are good models for investigating novel DNA damage repair mechanisms, because they exhibit a very low spontaneous mutation rate, although thriving at a temperature up to 80°C, a temperature at which spontaneous DNA lesions occur at a greatly accelerated rate (Grogan et al., 2001). In addition, DNA-strand-breaking agents can stimulate forward mutation in *S. acidocaldarius* (Reilly and Grogan, 2002), suggesting that the organism also shows mutagenic DNA damage tolerance. To date, the underlying mechanisms by which these crenarchaea undergo error-free as well as mutagenic DNA damage tolerance remain elusive. To address these questions, we conducted genetic analysis of all four DNA polymerase genes in *S. islandicus.* Our results have defined a novel TLS polymerase in the domain of Archaea, suggesting that different modes of DNA polymerase switches occur in DNA replication for the normal growth and upon encountering bulky DNA lesions in Sulfolobales. The proposed scheme of polymerase switches is summarized in Figure 7.

### Dpo1 Functions as the Replicative DNA Polymerase

In this model, Dpo1 is regarded as the replicative DNA polymerase in *S. islandicus*, and the following findings support the proposal. First, characterizations of the four *S. solfataricus* DNA polymerases have revealed two high-fidelity DNA polymerases in this archaeon, Dpo1 and Dpo3, and both DNA polymerases can function as the replicative polymerase because they both are full-length B-family DNA polymerases with the active proofreading from their exonuclease domains (Zhang et al., 2010; Bauer et al., 2012; Yan et al., 2017). Second, both enzymes exhibit enhanced processivity in the presence of proliferating cell nuclear antigen (PCNA)/replication factor C (RFC), the archaeal replication clamp, and its loader (Dionne et al., 2003; Choi et al., 2011). Nevertheless, genetic analysis has revealed that only *dpo1* gene is essential in *S. islandicus*, as shown here and in previous works (Martinez-Alvarez et al., 2017; Zhang et al., 2018). For this reason, if Dpo3 could be a component of the *S. islandicus* replication machinery as suggested for the *S. solfataricus* replisome (Yan et al., 2017), it should be a dispensable one.

### The Advantage of Dpo4 as the Housekeeping TLS Enzyme in Dealing With Accelerated Spontaneous Lesions

The *S. solfataricus* Dpo4 has served as an excellent model for studying TLS mechanisms of the Y-family DNA polymerases. By now, *in vivo* functions of the encoding genes have been investigated in three organisms, including *S solfataricus*, *S. acidocaldarius*, and *S. islandicus*. A genetic study of the *S. solfataricus dpo4* has revealed that Δ*dpo4* does not show any growth defect, but loss of *dpo4* has rendered the mutant more sensitive to cisplatin (Wong et al., 2010). Functional study of the *S. acidocaldarius dbh*, a *Ssodpo4* homolog, indicates that the encoded PolY plays an important role in the normal growth because the gene deletion mutant exhibits an elevated level of GC to TA transversions and a reduced level of frameshift mutations (Sakofsky et al., 2012). Here, our studies on the *S. islandicus dpo4* suggest that Dpo4 may mediate housekeeping TLS during DNA replication in the normal growth, consistent with the role of the *S. acidocaldarius dbh* gene in the normal growth, because these archaeal PolY enzymes are constitutively expressed to a high level both in *S. islandicus* (Supplementary Figure S1) and in *S. solfataricus* (Gruz et al., 2003; Gotz et al., 2007). All these data are compliant with a function for these PolY enzymes in chromosome duplication during normal growth at least for the Sulfolobales organisms (Figure 7).

Hyperthermophiles suffer from greatly accelerated levels of depurination and deamination even in the absence of any external mutagens (Lindahl and Nyberg, 1972) and from a much higher production of 7,8-dihydro-8-oxo-deoxyguanosine (8-oxodG) at their physiological growth conditions (Barbier et al., 2016; Killelea et al., 2019). These spontaneous DNA lesions would effectively block the *in vivo* progression of DNA replication by Dpo1, because the replicase stops upon encountering such DNA lesions *in vitro*, yielding stalled replication forks (Gruz et al., 2003; Choi et al., 2011). To avoid replication fork collapse, which could eventually lead to cell death, the stalled replication forks have to be restarted promptly, and this requires that a TLS polymerase be readily recruited to the damaged site for conducting lesion-bypass DNA synthesis. After the lesion bypass, Dpo1 takes over the DNA replication task again, resuming the normal DNA replication. Given that SsoDpo4 can bypass 8-oxodG in an error-free manner *in vitro* (Rechkoblit et al., 2006), the archaeal PolY is expected to play an important role in preventing GC to TA transversions during the normal growth. In addition, the Dpo4 is also proficient in the bypass of deaminated and depurinated products *in vitro* (Gruz et al., 2003; Fiala et al., 2007; Choi et al., 2011) and thus implicated in bypassing these spontaneous lesions *in vivo* in the normal growth. Taken together, these data have provided the basis arguing for the evolution of the archaeal PolY enzyme as the housekeeping TLS in Sulfolobales.

Genetic studies of *S. islandicus dpo4* have shown another important feature for this TLS polymerase: loss of gene induces ∼10% reduction in + 1 frameshift mutation, which typically occurred following mononucleotide runs of A (A4 or A5) (Supplementary Figure S10). Mechanistically, the *dpo4*-dependent + 1 frameshift mutations are to be yielded by the nascent strand slippage mechanism during DNA replication. However, this is in contrast to the biochemical characterization of SsoDpo4 in which this PolY enzyme mediates single-base deletion via the template slippage mechanism *in vitro* (Wu et al., 2011). Because the SisDpo4 shows >90% amino acid sequence identity to SsoDpo4, the apparent discrepancy between *in vivo* data obtained with the former and the *in vitro* data obtained with the latter suggests that the Dpo4-mediated TLS in Sulfolobales is subjected to regulation by complex mechanisms.

Further, the archaeal PolYs have maintained their function in damage-inducible DNA lesion bypass. We found that the *S. islandicus* Dpo4 may mediate error-free NQO-induced lesion bypasses *in vivo*, and this is consistent with the results obtained for the bacterial Pol IV and the eukaryotic Pol κ, the other DinB members of the Y-family DNA polymerases (Shen et al., 2002; Avkin et al., 2004; Yuan et al., 2008; Zhang and Guengerich, 2010; Jha et al., 2016). Therefore, the error-free bypass of N_2_-dG adduct by DinB family protein is conserved in all three domains of life.

### Dpo2 Is Responsible for Mutagen-Induced DNA Lesion Bypass

Our genetic analyses in *S. islandicus* show, for the first time, that Dpo2 is specialized in bypassing DNA lesions induced by external mutagens. Three lines of evidence support this conclusion: (a) western analysis shows that Dpo2 expression is correlated to the level of DNA damage in *S. islandicus*, (b) loss-of-function analysis reveals that *dpo2* is primarily responsible for DNA damage tolerance to helix-distorting lesions and that it is the only source of mutagenic DNA tolerance in this archaeon, and (c) the finding that constitutive high-level expression of Dpo2 in *S. islandicus* hardly affects the spontaneous mutation is incompliant with any housekeeping TLS activity for this DNA polymerase.

Dpo2 is 555 aa in size, which is much smaller than Dpo1, the replicative DNA polymerase (883 aa) (Guo et al., 2011). The archaeal Dpo2 lacks the 3′ → 5′ exonuclease domain that is otherwise present in the bacterial Pol II and the eukaryotic Pol ζ enzymes, the two other B-family TLS polymerases. In addition, Dpo2 also bears a substitution to the first aspartate in YGDTDS, the Pol C motif in the active center of B-family DNA polymerases (Supplementary Figure S5) (Bernad et al., 1990; Copeland and Wang, 1993; Kropp et al., 2017), and biochemical characterization of the *S. solfataricus* Dpo2 only detected a low primer extension activity (Choi et al., 2011). Consequently, Dpo2 was constantly predicted as an inactive polymerase (Rogozin et al., 2008; Makarova et al., 2014). However, our genetic analysis has shown that although dispensable for cell viability, Dpo2 facilitates cell survival and mediates targeted mutagenesis in *S. islandicus* in the presence of external mutagens. Therefore, the encoded enzyme must be active.

We reason that the activity of Dpo2 could be much more active than that observed for the *E. coli*-expressed *S. solfataricus* Dpo2, and two findings support this reasoning. First, the recombinant enzyme obtained from *E. coli* could be very different from the native enzyme. This is evident for the recombinant *S. solfataricus* Dpo2 employed for the previous research because the recombinant protein was readily inactivated at 50–60°C (Choi et al., 2011), a temperature that is well below the physiological growth condition of Sulfolobales (75–80°C) (Zillig et al., 1980). Indeed, in a seminal comparison between a recombinant esterase obtained from the mesophilic *E. coli* and that from the hyperthermophilic *S. islandicus*, major differences in activities between the two versions of the same enzyme were revealed (Mei et al., 2012). Second, Dpo2 is one of the cysteine-rich proteins in *S. islandicus* as it contains seven cysteine residues. Owing to a relative high level of redox condition in thermoacidophilic cells, most, if not all, cysteine residues of a protein are engaged in the formation of either intramolecular or intermolecular disulfide bonds in *S. islandicus* cells. For example, the *S. islandicus* esterase contains three cysteine residues that are involved in intramolecular disulfide bond formation (Stiefler-Jensen et al., 2017). Therefore, Sulfolobales Dpo2 proteins should be expressed from a Sulfolobales host, obtained from an *E. coli* host engineered for improved disulfide bond formation, and re-examined for their biochemical properties.

In addition, there are three ORFs in the *dpo2* gene locus, and these genes are well conserved in Sulfolobales genomes (Makarova et al., 2014). They could code for Dpo2-associated factors (Daf) and work in concert with Dpo2 in the TLS process. In addition, other DNA replication proteins, such as PCNA, RFC, and primases, should be studied for their possible interaction with the Sulfolobus-expressed Dpo2 to reveal additional Daf (Figure 7).

### Insights Into the Mechanisms of Dpo2-Mediated Lesion Bypass

Strikingly, all Dpo2-dependent hotspot mutations occur at either a guanine or a cytosine of targeted sites in the *apt* gene of *S. islandicus*, but no *dpo2*-dependent frameshifts are observed in this organism (Supplementary Figure S11). These results are in contrast to those obtained with the bacterial Pol II, which mainly mediates frameshift mutations in TLS using the lesion loop-out mechanism (Becherel et al., 2002; Wang and Yang, 2009). For the eukaryotic Pol ζ, the situation is more complex. This eukaryotic DNA polymerase is very efficient in elongation of primer termini that are positioned opposite the damaged base or non-instructional lesions such as UV dimers or apurinic/apyrimidinic sites (Johnson et al., 2000; Haracska et al., 2003), or mismatched primer termini generated by other polymerases such as Y-family TLS polymerases (Makarova and Burgers, 2015). In *S. islandicus*, Dpo2 is the only DNA polymerase that mediates UV tolerance and exerts both error-free and error-prone bypass of photoproduct during UV–lesion repair. This is in contrast to the yeast Pol ζ that plays a relatively minor role in UV-induced photoproduct bypass because the yeast Y-family Pol η plays a major role in enhancing UV resistance (McDonald et al., 1997).

A close examination of mutation spectra induced by three different mutagens has revealed interesting insights into the possible mechanisms of the Dpo2-mediated lesion bypass in *S. islandicus*. First, Dpo2-dependent mutation hotspot sites induced by NQO are located within TGGGA site in the WT strain in which G → A mutation occurs exclusively at the first two G, leading to amber and opal mutations that truncate the encoded protein (Supplementary Figure S11). Given NQO primarily induces N_2_-dG bulky adducts in DNA (Menichini et al., 1989), the GC → AT transition mutations by Dpo2 in NQO-treated *S. islandicus* cells require insertion of dT opposite the lesion in the TLS process. Second, accordingly, GC → TA transversions require incorporation of a dA opposite the cisplatin-induced damage at dG. Interestingly, at one of these mutation hotspots induced by cisplatin, both G → T and G → A mutations are detected, and all these mutations are dependent on Dpo2 (Supplementary Figure S11), indicating a complex mode of repair of cisplatin-induced lesions in *Sulfolobus*. Third, UV-induced Dpo2-dependent mutations include GC → AT transitions and GG → AA/CC → TT tandem mutations, which are exclusively occurred at dipyrimidine site (Supplementary Figure S11), given that bypass of cytosines in linked pyrimidine dimer would be error prone whereas bypass of TT dimer could be error free. These data suggest that in the TLS bypass of dipyrimidine sites, Dpo2 probably incorporates two dAs opposite the linked dimer in *S. islandicus* cells, leading to error-free bypass of TT-photoproducts and yielding mutations at CC or TC/CT photoproducts. Furthermore, although UV-induced GC → AT is also enriched at GGGA sites, the UV mutation hotspot sites are located at the third G, suggesting that bypass of TC photoproduct at the other strand contributes to these mutations. Indeed, 6-4 photoproduct (PP) at TC site represents the dominant form of this type of lesion in UV-irradiated DNA and very few photoproducts are formed at CT sites (Bourre et al., 1987; Friedberg et al., 2005), suggesting that bypass of 6-4 PP at TC sites is probably the main source of UV mutagenesis in *S. islandicus*. It would be very interesting to investigate how Dpo2 bypasses these bulky DNA lesions *in vitro*.

Investigation of DNA repair in Archaea has revealed that these organisms, crenarchaeota in particular, lack several classical DNA repair pathways that are well conserved in bacteria and eukaryotes, including nucleotide excision repair (NER) and canonical mismatch repair pathways that are very important for DNA damage repair (White and Allers, 2018). Nevertheless, they have evolved novel pathways to deal with various types of DNA damage. For example, Sulfolobales code for the UV-induced pilus (Frols et al., 2008; Ajon et al., 2011) and the crenarchaeotal DNA transfer system (van Wolferen et al., 2016), both of which work in concert to enable intercellular DNA transfer and facilitate DNA repair via homologous recombination. Here, we show that the *dpo2* gene is involved in mediating mutagenic DNA tolerance of helix-distortion lesions in Crenarchaeota, an archaeal lineage lacking the canonical NER pathway (White and Allers, 2018). In light of the recent finding that a euryarchaeal PolB is also implicated in DNA damage repair (Kushida et al., 2019), comparative studies of these archaeal PolB polymerases will yield further insights into the functions of these unusual archaeal PolBs in DNA repair and the involved mechanisms.

## Data Availability Statement

All data required to evaluate the conclusions of this study can be found in either the main text or the Supplementary Material.

## Author Contributions

XF, XL, RX, RZ, WF, JL, WH, and QS contributed to the acquisition of data and the revision of the manuscript. XF, WH, and QS interpreted the data. XF and QS designed the experiments and wrote the manuscript. All authors contributed to the article and approved the submitted version.

## Conflict of Interest

The authors declare that the research was conducted in the absence of any commercial or financial relationships that could be construed as a potential conflict of interest.
