# Supplementary material for: A Unique B-Family DNA Polymerase Facilitating Error-Prone DNA Damage Tolerance in Crenarchaeota
Source: Front Microbiol. 2020 Jul 23;11:1585. doi: 10.3389/fmicb.2020.01585 (PMC7390963; doi:10.3389/fmicb.2020.01585)
Supplement: Supplementary file 1 [file Data_Sheet_1.pdf]

Supplementary Material

Supplementary Tables

Supplementary Table S1. Strains and plasmids used in this study

**Strains**	**Genotype**	**Source**
E233S	Δ*pyrEF*Δ*lacS*	Deng *et al*., 2009 (Deng et al., 2009)
Δ*dpo2*	Δ*pyrEF*Δ*lacS*Δ*dpo2*	This work
Δ*dpo3*	Δ*pyrEF*Δ*lacS*Δ*dpo3*	This work
Δ*dpo4*	Δ*pyrEF*Δ*lacS*Δ*dpo4*	This work
**Plasmids**	**Features**	
pSeSD	A *Sulfolobus-E.coli* shuttle vector carrying an expression cassette controlled under a synthetic strong promoter ParaS-SD	Peng *et al*., 2012 (Peng et al., 2012)
pSeSD_*dpo2*	pSeSD carrying Dpo2 encoding sequence	This work
pSe-Rp	The plasmid contains a DNA fragment of two tandem copies of CRISPR repeat sequences for the construction of the artificial mini-CRISPR array	Peng *et al*., 2015 (Peng et al., 2015)
pAC-*dpo1*, *-2*, *-3*, *-4*	pSe-Rp carrying a spacer matching to the protospacer in the coding region of *dpo1*, *-2*, *-3*, *-4* gene correspondingly in genome	This work
pGE-*dpo1*, *-2*, *-3*, *-4*	The genome-editing plasmid derived from pAC-*dpo1*, *-2*, *-3*, *-4* respectively, with the corresponding	This work

	donor DNA inserted between SphI and XholI	

Supplementary Table S2. Oligos used in this study

Oligos	Sequence
**Construction of *dpo* mutant**	
KO*dpo1*Larm-F	tttgcatgcCATAGAATTGAATAAGGAGCTTCTGG
KO*dpo1*SOE-R	CCTCCTGTGAAAGGAATATCAAATAAGGTAAGTTGCT
KO*dpo1*SOE-F	TTGATATTCCTTTCACAGGAGGAAAAGGGAATAATTAA
KO*dpo1*Rarm-R	tttctcgagTGGAAATATGAACCATACAGTGTATC
KO*dpo1*spf	aagTTATTGGGTATATCAAAAGTTAAGGTGGATACGCTAATAT
KO*dpo1*spr	agcATATTAGCGTATCCACCTTAACTTTTGATATACCCAATAA
KO*dpo2* F	AGGATTTAGGGGATTGGA
KO*dpo2* R	TGGAGGGGAACCATCGCC
dpo2 inner F	CCCACTTCACGAGATAGCCT
dpo2 inner R	CCTCCCTTATCCGCCATCAT
KO*dpo2*Larm-F	tttgcatgcGGAAGAACACAGCCATCATACA
KO*dpo2*SOE-R	CCCTCCTCAAGTACTCCTCCATTTCTCGCATTCCTC
KO*dpo2*SOE-F	GAAATGGAGGAGTACTTGAGGAGGGTTTATGAC
KO*dpo2*Rarm-R	tttctcgagACCTCCATCATCACTTACTTTA
KO*dpo2*spf	aagTTGATTGTAAAATACAACATTTCAGCTGAAACCGTTGACG
KO*dpo2*spr	agcCGTCAACGGTTTCAGCTGAAATGTTGTATTTTACAATCAA
KO*dpo3* F	CTAGTGGCCGATGATACGCT

KO*dpo3* R	TGAGAAAGTTCAAGTGCGAGA
dpo3 inner F	TTCTTTCGCACTATGAGGGT
dpo3 inner R	AGATCATCCATGCTTTCGTCT
KO*dpo3*Larm-F	tttgcatgcCATGCATGCTCCGAGAGTATCTTTATCCCT
KO*dpo3*SOE-R	ATTTCTTCTTAGAACTAACCAAATGACTGGCT
KO*dpo3*SOE-F	TCATTTGGTTAGTTCTAAGAAGAAATAATGTCAGTAAA
KO*dpo3*Rarm-R	tttctcgagCCGCTCGAGTTAGACAGGATTGAGACTGC
KO*dpo3*spf	aagCTAATTTACATTTGGAGCATTGATGATGAAGGTAACAGTT
KO*dpo3*spr	agcAACTGTTACCTTCATCATCAATGCTCCAAATGTAAATTAG
KO*dpo4* F	CTCTCTCTCCCAGCGAATCAG
KO*dpo4* R	ATGGGCAAGAAAGGGGCAAA
dpo4 inner F	ATGGCAAAGCCAAATGGGAT
dpo4 inner R	TGGCTTTAGCCTCACCAATTA
KO*dpo4*Larm-F	tttgcatgcCATGCATGCTATCACTTCTCCTCCACCTT
KO*dpo4*SOE-R	CTAAACCTTACTCCGCGTAAAAGTAGTCAAAATCAACGA
KO*dpo4*SOE-F	GACTACTTTTACGCGGAGTAAGGTTTAGCAAATTCATC
KO*dpo4*Rarm-R	tttctcgagCCGCTCGAGCATGTGATGAAGACCTTTGG
KO*dpo4*spf	aagATAGTTGAAGCAAAGAAAATTTTACCTAATGCAGTTTACT
KO*dpo4*spr	agcAGTAAACTGCATTAGGTAAAATTTTCTTTGCTTCAACTAT

**Overexpression of *dpo2***	
dpo2-NdeI-F	TCCACTcatatgCGAGAAATGGAGGAGTACGT
dpo2-SalI-R	ATTTgtcgacACATCTAGAGATCACCTCT
**Others**	
Sisapt-F	TACCCGGATCATATAACCCAG
Sisapt-R	AAGGTTTTTGTGGTTGGTGAT

Supplementary Table S3. Dpo2 homologues in crenarchaeal species

**Dpo2 Homologue**	**Species**	**Size (aa)**	**Identity to SisDpo2 (%)**	**Similarity to SisDpo2 (%)**
Sis	*Sulfolobus islandicus* Rey15A	555	100	100
Sso	*Sulfolobus solfataricus* P2	561	91	96
Sto	*Sulfolobus tokodaii* str. 7	540	68	81
Sac	*Sulfolobus acidocaldarius* DSM639	582	54	72
Ahos	*Acidianus hospitalis* W1	554	56	73
Mese	*Metallosphaera sedula* DSM5348	562	53	71
Mecu	*Metallosphaera cuprina* AR-4	562	53	73
Ffo	*Fervidicoccus fontis* Kam940	541	31	52
Calag	*Caldisphaera lagunensis* DSM 15908	624	29	46
Aca	*Aeropyrum camini* SY1	636	30	47
Ape	*Aeropyrum pernix* K1	633	32	48
Tagg	*Thermosphaera aggregans* DSM 11486	636	32	51
Tcal	*Thermogladius calderae* 1633	644	29	45
Smar	*Staphylothermus marinus* M1	648	34	55
Shell	*Staphylothermus hellenicus* DSM 12710	648	36	59

Supplementary Figures

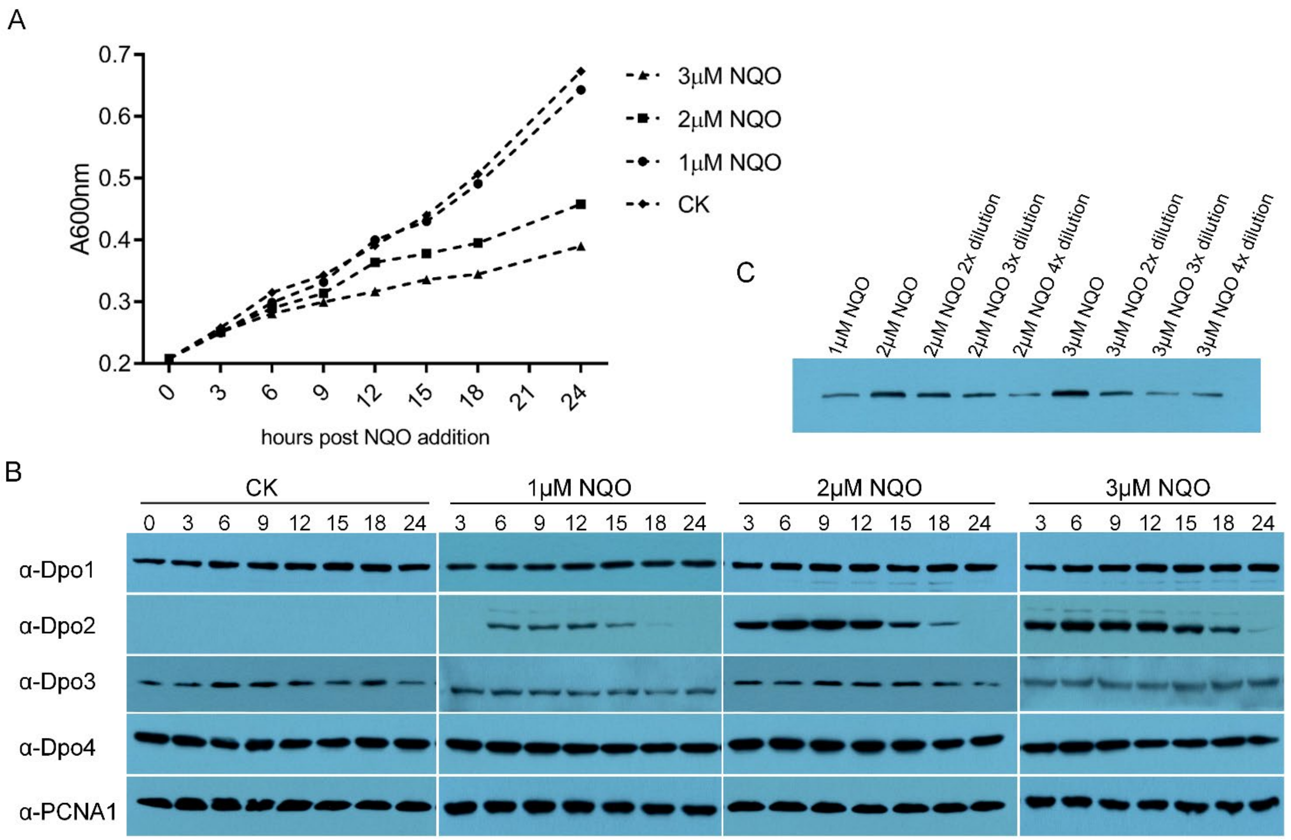

Supplementary Figure S1. Effect of NQO on cell growth and expression of DNA polymerases in *S. islandicus*

(A) Growth curve of the wild-type strain of *S. islandicus* E233S in the presence of NQO. NQO was added to exponetial growth cultures (A600nm=0.2) at the concentration of 0, 1, 2 and 3 μM, and incubated for 24 h during which cell samples were taken for monitoring their A600 values.
(B) Expression profiles of the four DNA polymerases revealed by western analysis. 10 μg of total cell extracts of NQO-treated samples (1, 2, 3 μM) and the untreated reference (CK) were used for the immunoblotting analysis using antibodies against each DNA polymerase. PCNA1, which has a constant expression upon DNA damage, serves as a loading control.
(C) Quantification of relative amounts of Dpo2 in samples taken from cultures incubated with different concentrations of NQO.

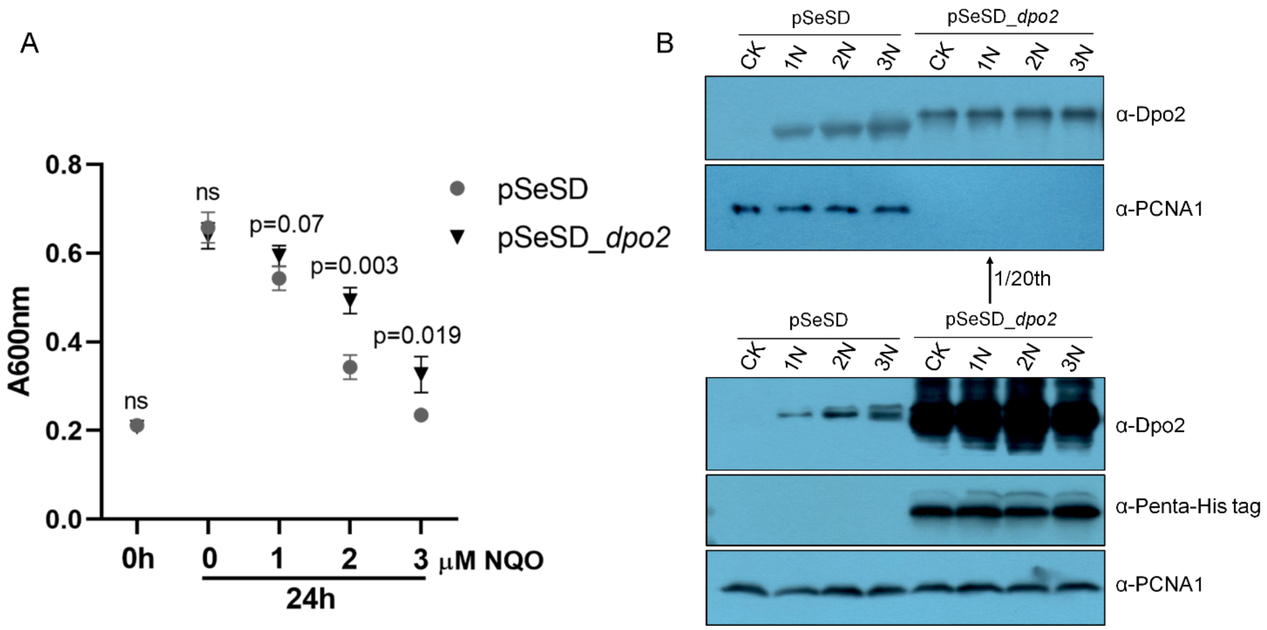

Supplementary Figure S2. Cell growth and western blotting analysis of *dpo2*-overexpression strain and its reference

(A) Exponentially growing cultures (A600nm=0.2) were incubated with 0, 1, 2 and 3 μM NQO for 24 hours. The A600nm value of each culture at 0h and 24 hour after NQO addition was plotted. Three independent experiments were performed with the standard deviation shown in the error bar. Unpaired t test was performed for each group of data, with p values indicated in the graph.
(B) Cell samples were taken at 6 hours after NQO addition and cell extracts were obtained by sonication and centrifugation. 10 μg of cell extracts of NQO-treated and control (CK) samples were used for immunoblotting analysis using Dpo2 and Penta-His Tag antibodies. To estimate the relative amounts of overexpressed Dpo2, samples of overexpression strain were diluted by 20 times individually and used for the western blot analysis. PCNA1 serves as an internal control. 1N, 2N and 3N refers to the sample incubated with 1, 2 and 3 μM NQO respectively.

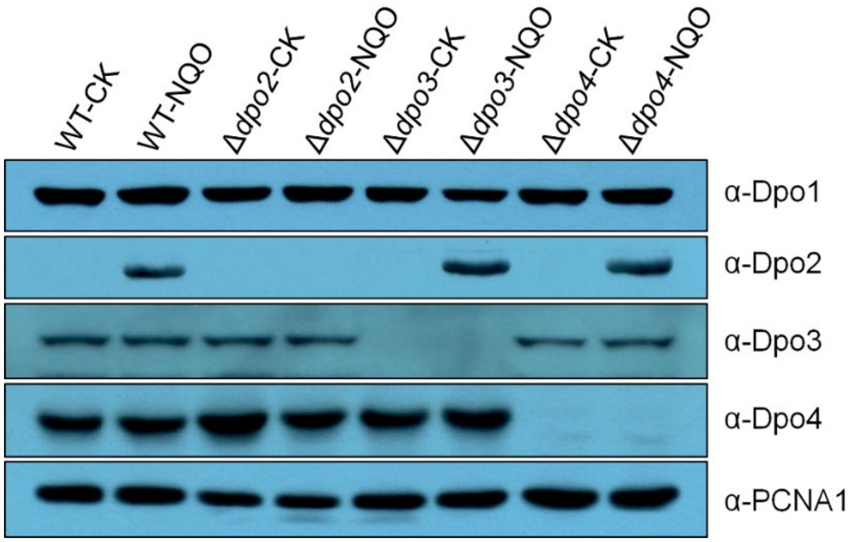

Supplementary Figure S3. Expression of each DNA polymerase in different strains upon NQO treatment

Exponentially growing cultures (A600nm=0.2) were incubated with 2 μM NQO for 6 hours and samples were taken for the preparation of cell extracts. Euqal amount of cell extracts (10 μg) for each sample were used in western blotting assay using antibodies against each DNA polymerase. PCNA1 serves as an internal control.

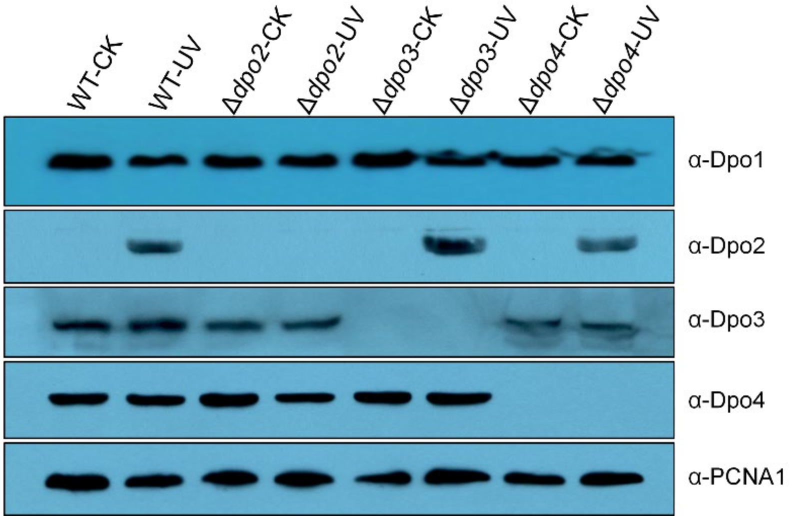

Supplementary Figure S4. Expression of each DNA polymerase in different strains post UV irradiation

Exponentially growing cultures (A600nm=0.2) were exposed to 50 J/m^2 UV-C light, then, the treaed cultures were allowed to recover for 6 h under the dark condition with shaking. Cell extracts were prepared and euqal amounts of cell extracts (10 μg) for each sample were used for the western blotting assay using antibodies against each DNA polymerase. PCNA1 serves as an internal control.

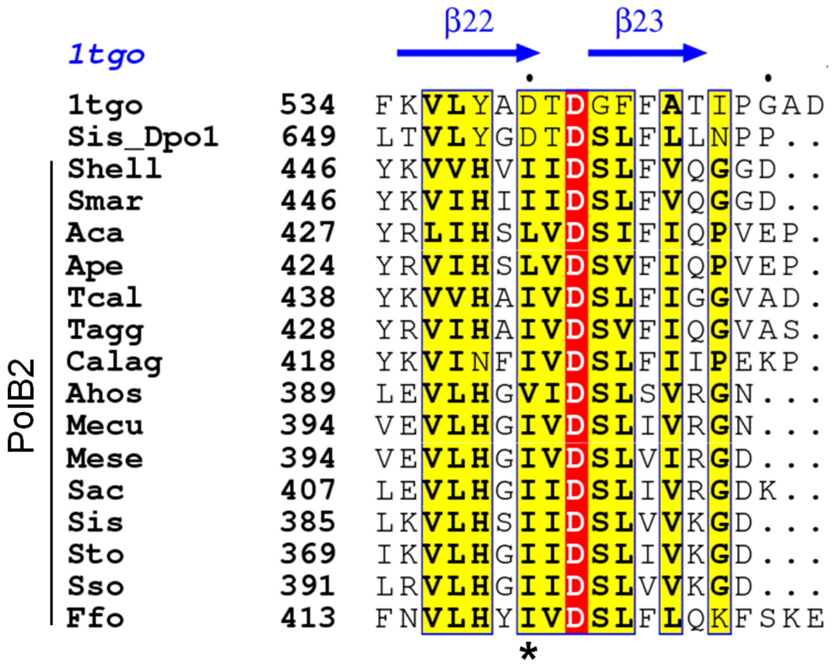

Supplementary Figure S5. Dpo2 homologues carry a substitution at the PolC motif

Structure-based sequence alignment of the PolC motif of Dpo2 homologues. The mutated aspartate in the PolC motif (YGDTDS) was indicated by the asterisk symbol. The structure of *Thermococcus gorgonarius* PolB (1tgo) was used as the template for the structure-based sequence alignment, which was performed using PROMALS3D webserver (Pei et al., 2008) and depicted using Espript 3 (Robert and Gouet, 2014). SisDpo1 harboring the canonical PolC motif was shown as the control.

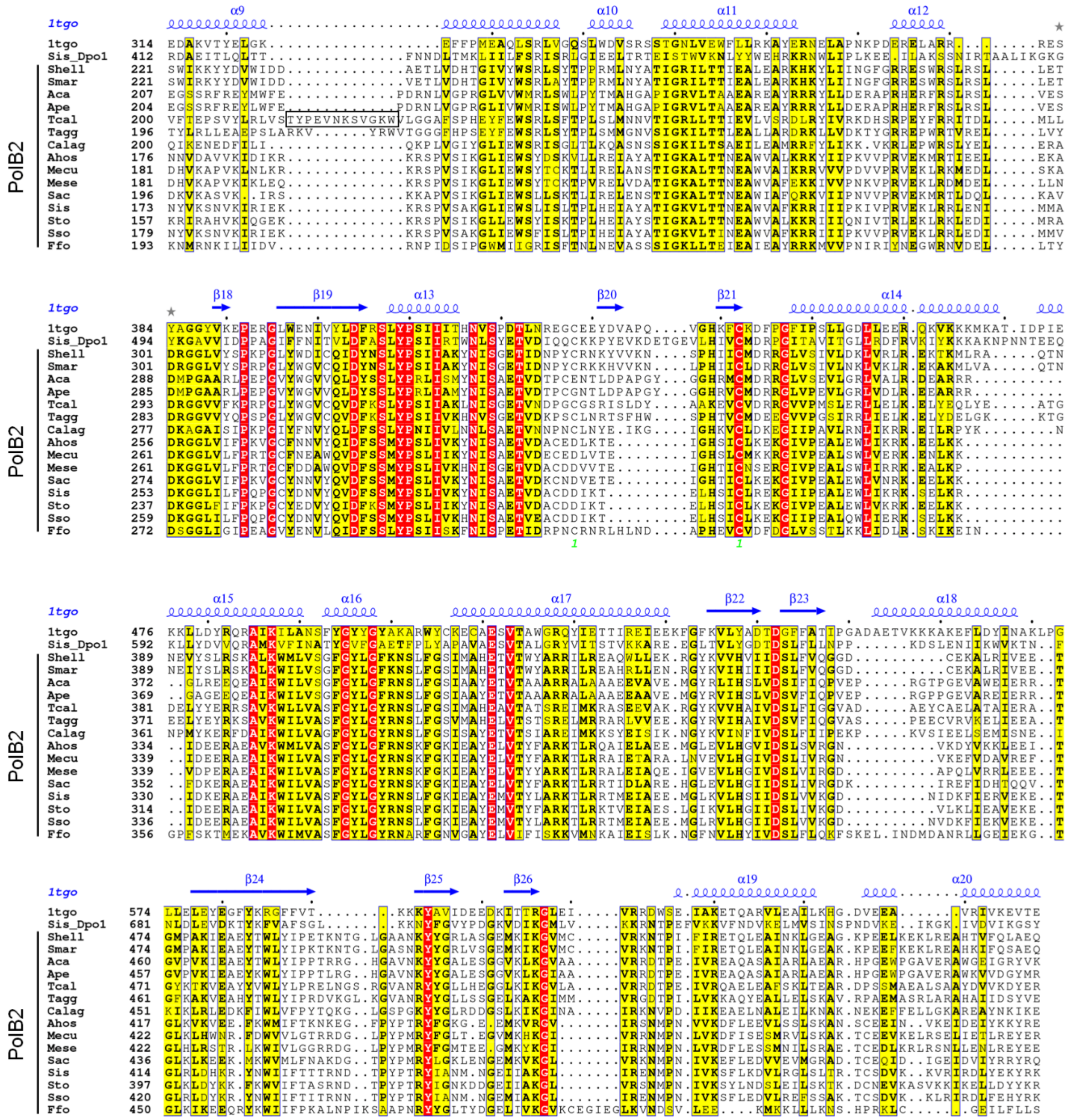

Supplementary Figure S6. Sequence alignment of conserved regions of Dpo2 homologues

The structure of *Thermococcus gorgonarius* PolB (1tgo) was used as the template for the structure-based sequence alignment, which was performed using PROMALS3D webserver (Pei et al., 2008) and depicted by Espript 3 (Robert and Gouet, 2014). SisDpo1 belonging to PolB1 family was shown as a control. Framed sequences indicates a 12 aa sequence insertion in *Thermogladius calderae* PolB2 (Tcal).

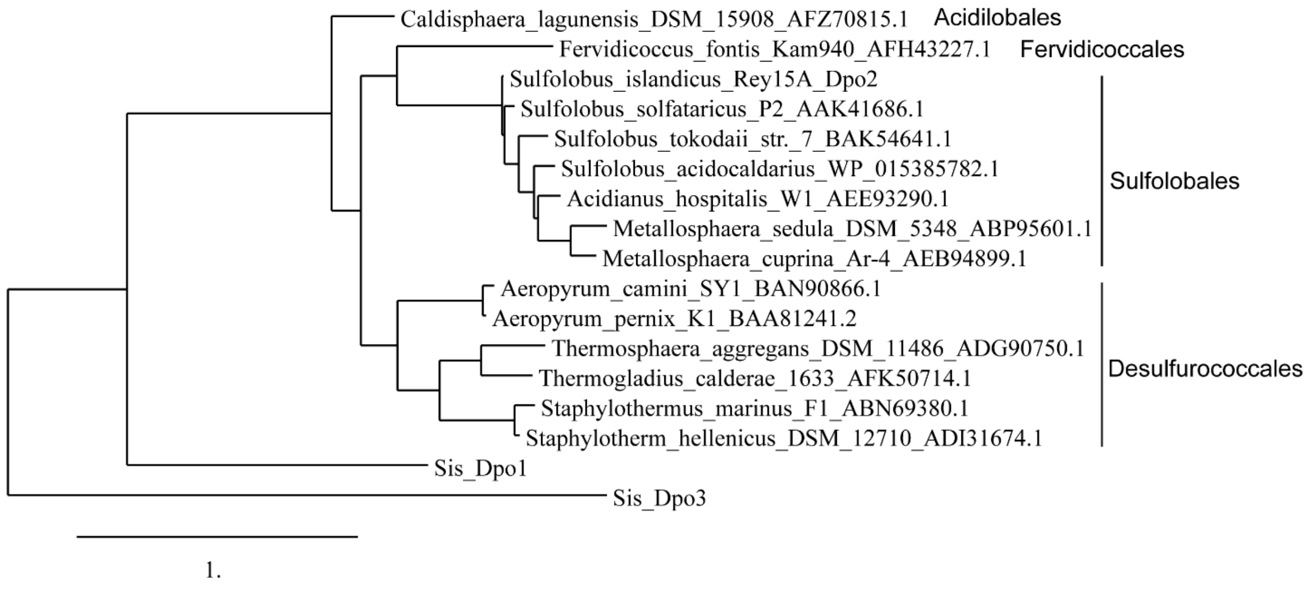

Supplementary Figure S7. Phylogenetic tree of Dpo2 homologues

The tree was constructed using sequences of Dpo2 homologs extracted from NCBI. These sequences were first aligned using MUSCLE, then the poorly aligned regions were removed by Gblocks program (v0.91b) using the default setting. The phylogenetic tree was constructed using the trimmed sequences with the PhyML program (v3.0) and the tree was visualized by using the TreeDyn program (v198.3). Sis_Dpo1 and Sis_Dpo3 sequences are used as the outgroup.

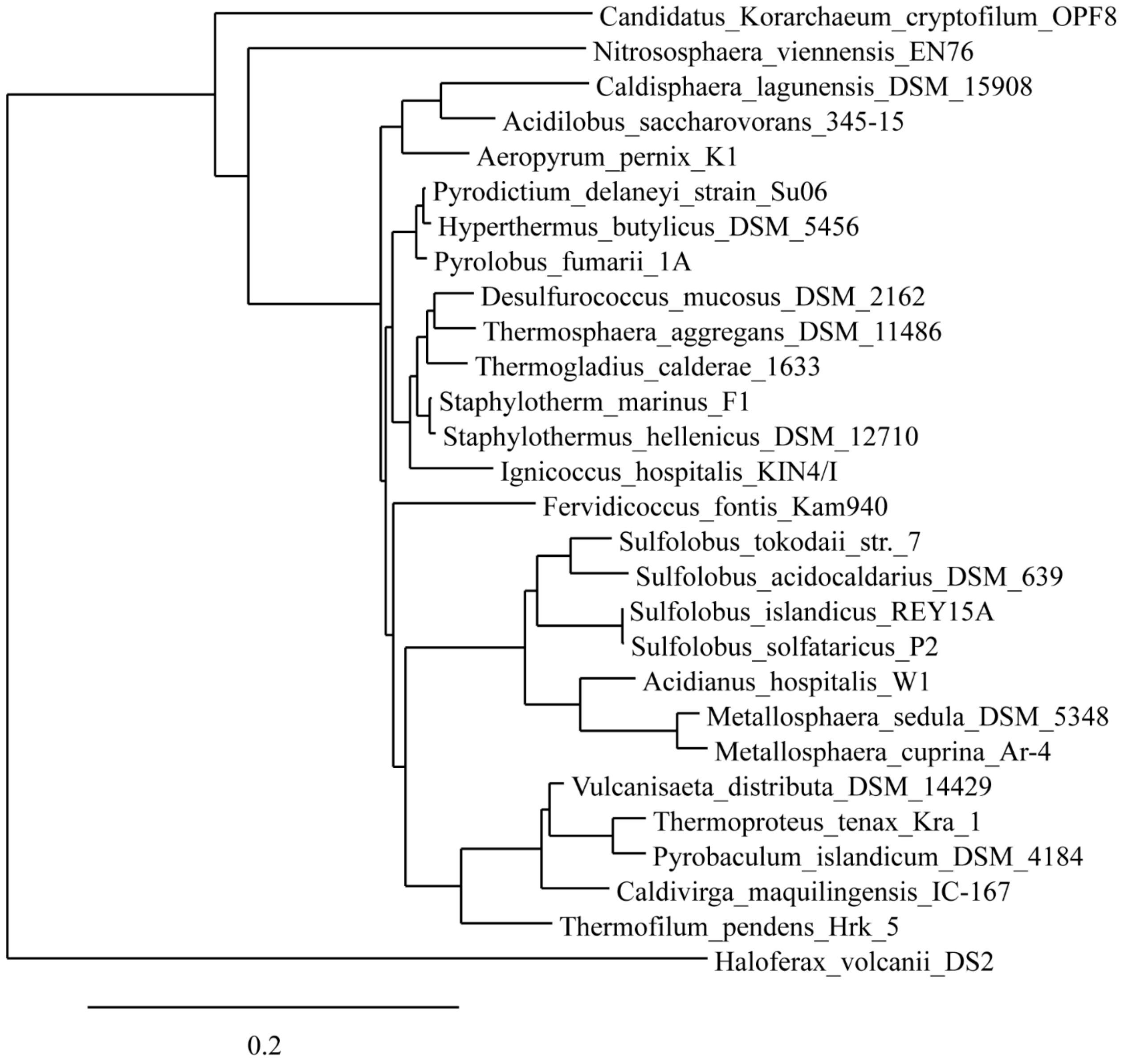

Supplementary Figure S8. Phylogenetic tree of crenarchaeal species

Phylogenetic trees of representative crenarchaeal species were constructed using their 16S rDNA sequences retrieved from the NCBI databases. The 16S rDNA sequences were first aligned using MUSCLE program, then, poorly aligned regions were removed by Gblocks program (v0.91b) using the default setting. The phylogenetic tree was constructed using the trimmed sequences with the PhyML program (v3.0) and visualized by the TreeDyn program (v198.3). The 16s rDNA sequence of *Haloferax volcanii* DS2 was used as the outgroup.

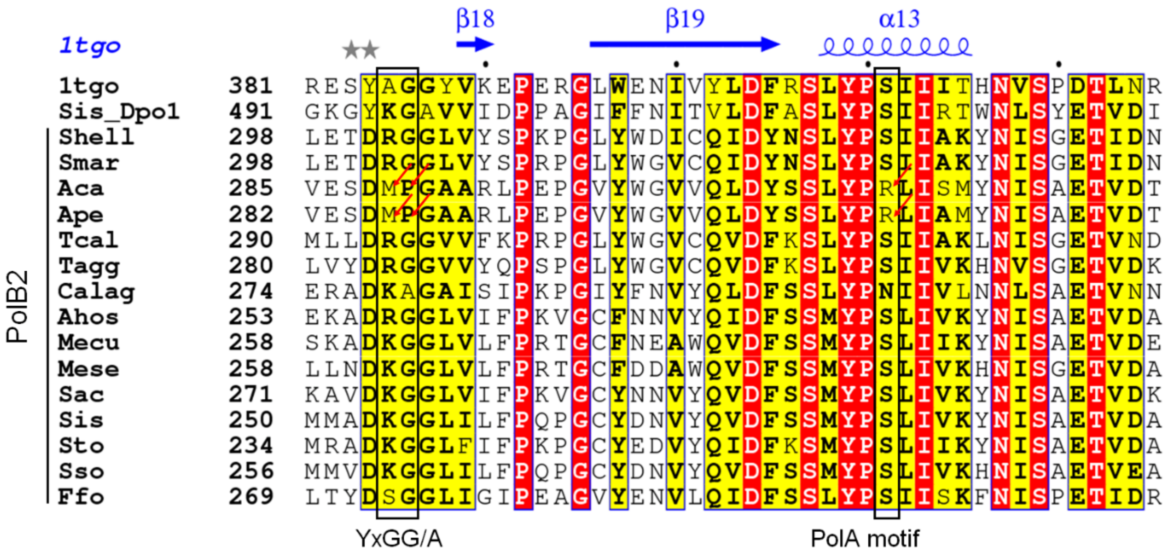

Supplementary Figure S9. Sequence alignment of YxGG/A and PolA motif of archaeal PolB2 homologs

The structure of *Thermococcus gorgonarius* PolB (1tgo) was used as the template to conduct structure-based sequence allignments using PROMALS3D webserver (Pei et al., 2008), and the resulting data were depicted by Esprit 3 (Robert and Gouet, 2014). Conserved residues are highlighted by yellow background and identical ones are in the red. Variations in the YxGG/A and PolA motifs of PolB2s from *Aeropyrum pernix K1* (Ape) and *Aeropyrum camini* SY1 are framed and indicated by red arrows.

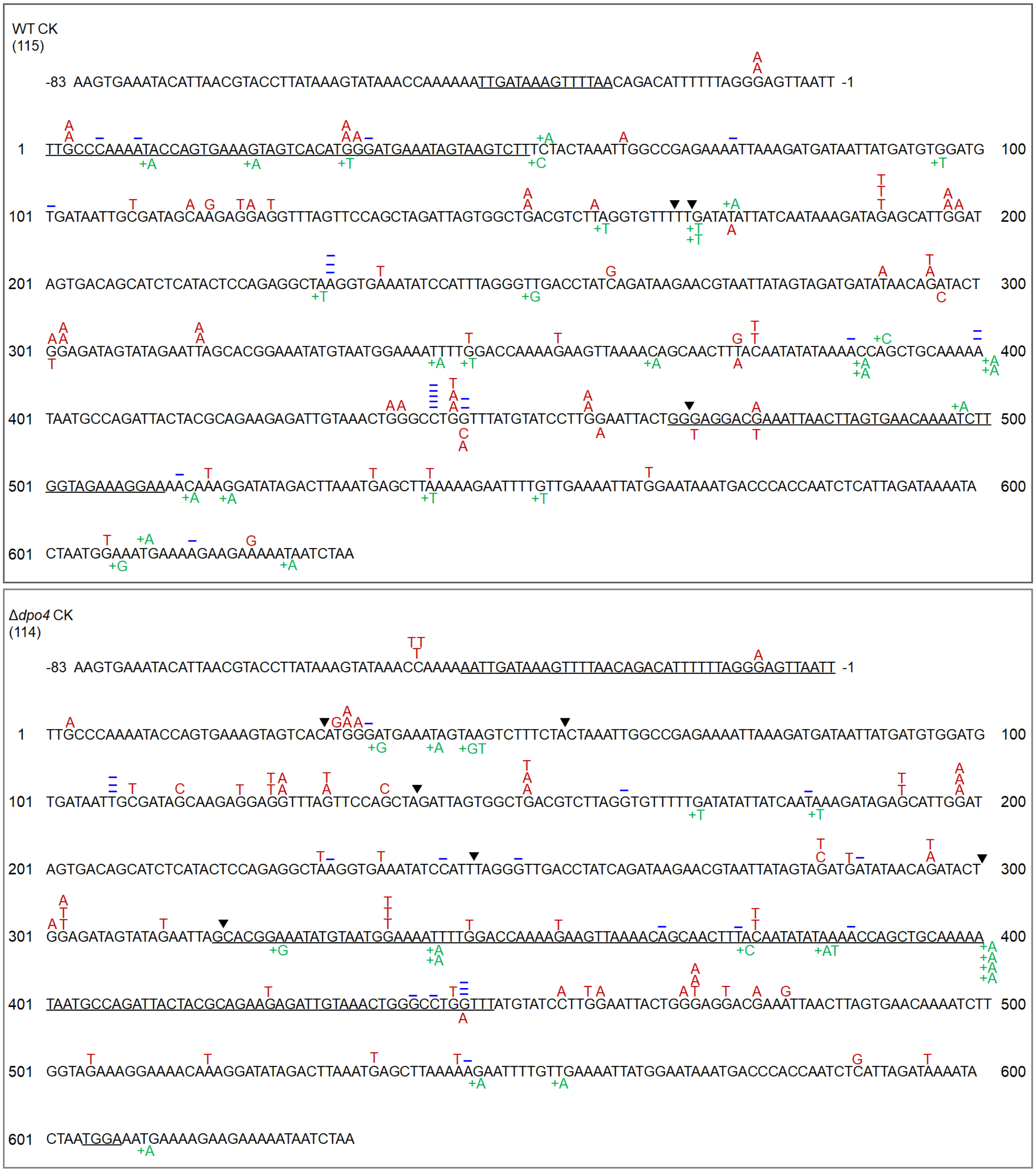

Supplementary Figure S10. Spontaneous mutation spectra of *apt3* locus in WT and Δ*dpo4*

Mutated bases are shown in red on the top of original ones. Single base deletions are indicated with blue "-" signs above the deleted bases, and single base insertions are indicated by green "+" signs beneath the bases immediately before the insertion positions with the inserted shown in green. Large insertions (>2bp) are shown with black triangle signs. Numbers in the bracket indicate the sample size (total number of analyzed mutants).

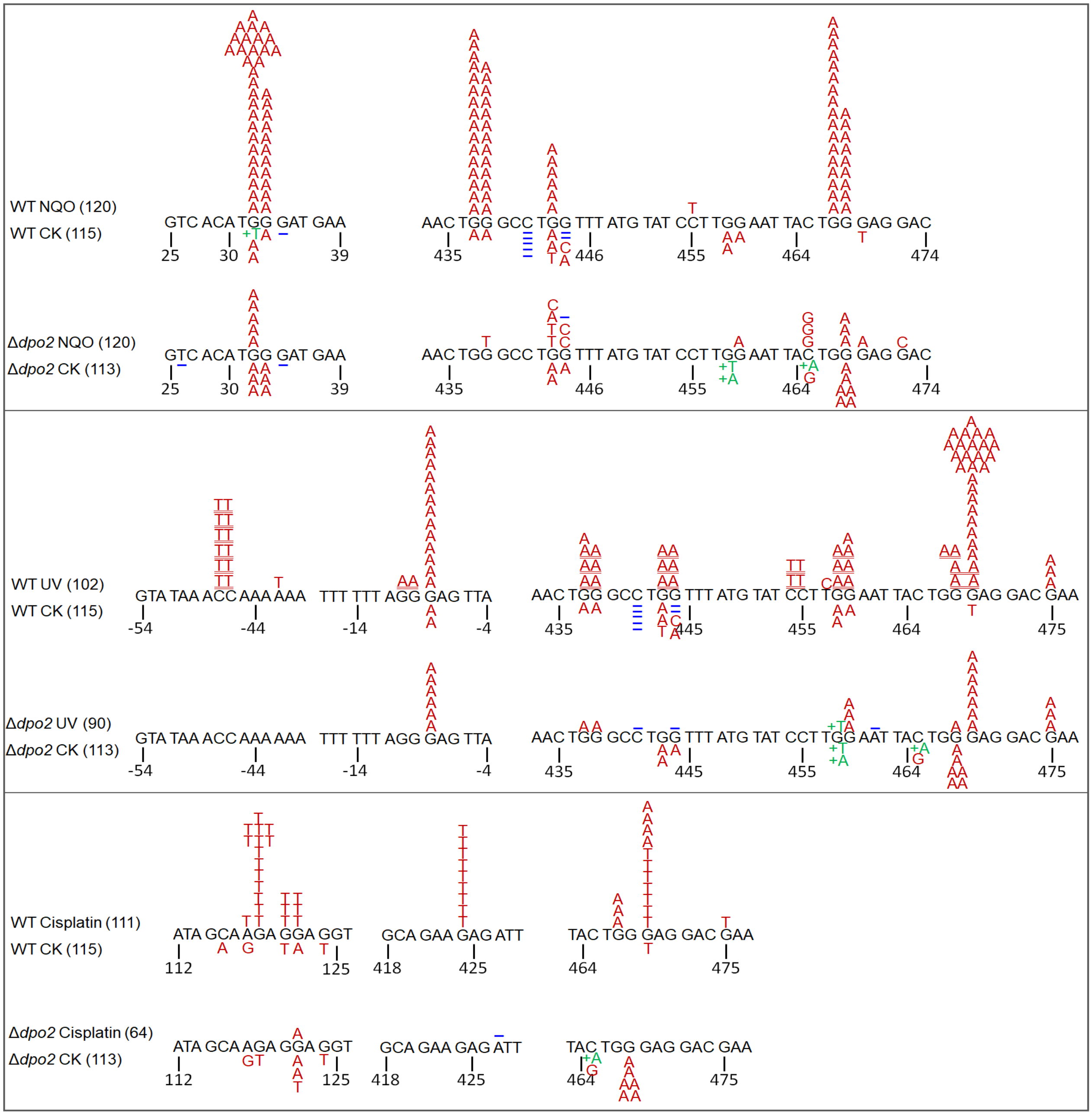

Supplementary Figure S11. DNA damage-induced mutation hotspots in the *apt3* locus

Only mutation hotspots are shown with their locations in the *apt3* locus indicated. The mutated bases of treated and reference samples are shown on the top of and under the original ones respectively. Tandem mutations are double underlined. Single base deletions are indicated with blue "-" signs above/beneath the deleted bases, and single base insertions are indicated by green "+" signs above/beneath the bases immediately before the insertion positions with the inserted shown in green. Large insertions (>2bp) are shown with black triangle signs. Numbers in the bracket indicate the sample size (total number of analyzed mutants).

Deng, L., Zhu, H., Chen, Z., Liang, Y.X., and She, Q. (2009). Unmarked gene deletion and host-vector system for the hyperthermophilic crenarchaeon Sulfolobus islandicus. *Extremophiles* 13(4)**,** 735-746. doi: 10.1007/s00792-009-0254-2.

Pei, J., Tang, M., and Grishin, N.V. (2008). PROMALS3D web server for accurate multiple protein sequence and structure alignments. *Nucleic Acids Res* 36(Web Server issue)**,** W30-34. doi: 10.1093/nar/gkn322.

Peng, N., Deng, L., Mei, Y., Jiang, D., Hu, Y., Awayez, M., et al. (2012). A synthetic arabinose-inducible promoter confers high levels of recombinant protein expression in hyperthermophilic archaeon Sulfolobus islandicus. *Appl Environ Microbiol* 78(16)**,** 5630-5637. doi: 10.1128/AEM.00855-12.

Peng, W., Feng, M., Feng, X., Liang, Y.X., and She, Q. (2015). An archaeal CRISPR type III-B system exhibiting distinctive RNA targeting features and mediating dual RNA and DNA interference. *Nucleic Acids Res* 43(1)**,** 406-417. doi: 10.1093/nar/gku1302.

Robert, X., and Gouet, P. (2014). Deciphering key features in protein structures with the new ENDscript server. *Nucleic Acids Res* 42(Web Server issue)**,** W320-324. doi: 10.1093/nar/gku316.
